# Differential dynamics and impacts of prophages and plasmids on the pangenome and virulence factor repertoires of Shiga toxin-producing *Escherichia coli* O145:H28

**DOI:** 10.1099/mgen.0.000323

**Published:** 2020-01-14

**Authors:** Keiji Nakamura, Kazunori Murase, Mitsuhiko P. Sato, Atsushi Toyoda, Takehiko Itoh, Jacques Georges Mainil, Denis Piérard, Shuji Yoshino, Keiko Kimata, Junko Isobe, Kazuko Seto, Yoshiki Etoh, Hiroshi Narimatsu, Shioko Saito, Jun Yatsuyanagi, Kenichi Lee, Sunao Iyoda, Makoto Ohnishi, Tadasuke Ooka, Yasuhiro Gotoh, Yoshitoshi Ogura, Tetsuya Hayashi

**Affiliations:** ^1^​ Graduate School of Medical Sciences, Kyushu University, Fukuoka, Japan; ^2^​ Faculty of Medicine, University of Miyazaki, Miyazaki, Japan; ^3^​ Center for Information Biology, National Institute of Genetics, Tokyo, Japan; ^4^​ Graduate School of Bioscience and Biotechnology, Tokyo Institute of Technology, Tokyo, Japan; ^5^​ Faculty of Veterinary Medicine, University of Liege, Liege, Belgium; ^6^​ Universitair Ziekenhuis Brussel, Brussels, Belgium; ^7^​ Miyazaki Prefectural Institute for Public Health and Environment, Miyazaki, Japan; ^8^​ Toyama Institute of Health, Toyama, Japan; ^9^​ Osaka Institute of Public Health, Osaka, Japan; ^10^​ Fukuoka Institute of Health and Environmental Sciences, Fukuoka, Japan; ^11^​ Oita Prefectural Institute of Health and Environment, Oita, Japan; ^12^​ Akita Research Center for Public Health and Environment, Akita, Japan; ^13^​ National Institute of Infectious Diseases, Tokyo, Japan; ^14^​ Graduate School of Medical and Dental Sciences, Kagoshima University, Kagoshima, Japan

**Keywords:** comparative genomics, complete genome, draft genome set, prophage and plasmid dynamics, Shiga toxin-producing *Escherichia coli*

## Abstract

Phages and plasmids play important roles in bacterial evolution and diversification. Although many draft genomes have been generated, phage and plasmid genomes are usually fragmented, limiting our understanding of their dynamics. Here, we performed a systematic analysis of 239 draft genomes and 7 complete genomes of Shiga toxin (Stx)-producing *Escherichia coli* O145:H28, the major virulence factors of which are encoded by prophages (PPs) or plasmids. The results indicated that PPs are more stably maintained than plasmids. A set of ancestrally acquired PPs was well conserved, while various PPs, including Stx phages, were acquired by multiple sublineages. In contrast, gains and losses of a wide range of plasmids have frequently occurred across the O145:H28 lineage, and only the virulence plasmid was well conserved. The different dynamics of PPs and plasmids have differentially impacted the pangenome of O145:H28, with high proportions of PP- and plasmid-associated genes in the variably present and rare gene fractions, respectively. The dynamics of PPs and plasmids have also strongly impacted virulence gene repertoires, such as the highly variable distribution of *stx* genes and the high conservation of a set of type III secretion effectors, which probably represents the core effectors of O145:H28 and the genes on the virulence plasmid in the entire O145:H28 population. These results provide detailed insights into the dynamics of PPs and plasmids, and show the application of genomic analyses using a large set of draft genomes and appropriately selected complete genomes.

## Data Summary

The raw read sequences and assembled scaffold sequences obtained in this study have been deposited in GenBank/EMBL/DDBJ under the BioProject accession number PRJDB8147. Six supplementary tables and four supplementary figures are available with the online version of this article.

**Impact Statement**

Various virulence determinants are transferred to pathogenic bacteria through horizontal gene transfer mediated by mobile genetic elements. Shiga toxin-producing *Escherichia coli* (STEC) strains cause severe human diseases, and most of their major virulence-associated genes are encoded by phages and plasmids. Although O157:H7 is the most predominant serotype, STEC includes strains of other serotypes that cause severe diseases. Highly variable prophage (PP) contents have been shown for STEC O157, and such variations are considered as the major driver generating the genetic diversification of STEC O157. However, the dynamics of PPs have not been analysed in non-O157 STEC, and for plasmids remain unknown even in O157 STEC. In this study, we present the results of a comprehensive analysis of STEC O145:H28, one of the major non-O157 STEC lineages. Detailed analysis of seven complete genomes in combination with a large draft genome set revealed not only the global population structure of O145:H28 but also the differential dynamics and impacts of PPs and plasmids on the pangenome structure and virulence factor repertoire. This study shows the importance and application of the combined analysis of a large set of draft genomes and appropriately selected complete genomes.

## Introduction

Advances in genome sequencing have revealed that various virulence determinants have been transferred to pathogenic bacteria through horizontal gene transfer mediated by mobile genetic elements (MGEs), such as plasmids, bacteriophages (or phages), transposons and integrative elements (IEs) [1, 2]. Despite the large number of bacterial genomes that have been sequenced, most are draft sequences in which plasmid and phage genomes are usually fragmented, limiting our understanding of the dynamics of these MGEs during bacterial genome evolution/diversification. Due to the recent advent of long-read sequencing technology, obtaining complete bacterial genomes has become easier. The use of multiple complete genomes and a large set of draft genomes would provide unprecedented opportunities to perform detailed analyses of the dynamics of plasmids and phages in bacteria of interest; however, such analyses have rarely been attempted and remain challenging.

Shiga toxin (Stx)-producing *Escherichia coli* (STEC) cause haemorrhagic colitis and haemolytic-uremic syndrome, and their major virulence factors (VFs) are encoded by MGEs [3–5]. Stxs are classified into Stx1 and Stx2. Although both types include several subtypes [6], all types are encoded by prophages (PPs). Another essential VF of major STEC strains is the type III secretion system (T3SS). The secretion apparatus is encoded by an IE called the locus of enterocyte effacement (LEE). Several T3SS effectors are encoded by the LEE, but more than 30 effectors (non-LEE effectors) are also encoded by multiple PPs [7–9]. Highly variable PP content has been detected even among STEC strains belonging to the same serogroup, and such variation is considered one of the major drivers of the genetic diversification of each STEC lineage [10–13]. However, although the genetic diversity of PPs has been well analysed in STEC O157:H7 [13, 14], it has not yet been systematically analysed in other STEC serotypes. Details of the variation in plasmid repertoires also remain unknown even in O157:H7, although most major STEC strains carry a large virulence plasmid (Vir plasmid) encoding enterohaemolysin and other potential VFs, and often contain one or more additional plasmids [nonvirulence plasmids (NV) plasmids] [9, 15, 16].

Among the many STEC serotypes, O157:H7 is the most prevalent worldwide, but strains of other serogroups also cause outbreaks and sporadic cases of infection. O145:H28 is one of the major non-O157 STEC lineages that causes severe diseases and is frequently isolated from humans, animals and foods [17–19]. In the recent surveillance and monitoring of STEC in Europe and the USA, up to 30–50 % of confirmed human STEC infections were caused by O157, and the proportion of O145 ranged from 2 to 4 % [20, 21]. However, many cases of severe illness, such as haemolytic-uremic syndrome, caused by O145:H28 infection have been reported worldwide [22–24]. Two O145:H28 strains have been completely sequenced [25], and the population structure has been examined by multilocus sequence typing (MLST) [26]. However, whole-genome sequence (WGS)-based phylogenetic analysis of O145:H28 has not been conducted and, thus, its global phylogeny and genetic diversity, including variations in MGEs, remain unexplored. Here, we performed a WGS-based phylogenetic analysis of 239 O145:H28 strains and in-depth analyses of the PPs and plasmids of 7 complete genomes. We further investigated the variations in PPs and plasmids and their impact on the pangenome of O145:H28. Our results reveal the highly dynamic features of PPs and plasmids in the diversification of this STEC lineage, and the differential dynamics and impacts of these MGEs on the pangenome and VF repertoire among O145:H28 strains.

## Methods

### Bacterial strains

The strains sequenced in this study were clinical isolates obtained in Japan and Belgium from 1998 to 2016, with the exception of 16 and 2 strains from asymptomatic carriers and foods, respectively. From the National Center for Biotechnology Information database (final accession, 12 October 2018), the read or assembled sequence data of O145:H28 were downloaded and included in the data set after confirming their H-genotypes using the EcOH database [27]. Low-quality sequences were excluded (coverage depth <30×, N50 contig length <40 kb or contamination determined by CheckM [28] >5 %), and strains were deduplicated if the recombination-free core sequences were identical. Finally, 239 strains (99 sequenced in this study and 140 from the database) were analysed (listed in Table S1, available with the online version of this article).

### Genome sequencing, assembly and annotation

The strains were grown in lysogeny broth at 37 °C. The purification of genomic DNA, the preparation of sequencing libraries, Illumina sequencing and sequence assembly were performed as previously described [29]. Strain 10942 was sequenced using Roche 454 GS FLX and an 8 kb mate-pair (MP) library, and the Illumina paired-end reads and 454 MP reads were assembled with Newbler (Roche); gap-filling was subsequently performed by PCR and Sanger sequencing of the PCR products. Strains 122715 and 112648 were sequenced using PacBio RS II, and sequence assembly and error correction were performed as previously described [30]. The complete genomes determined in this study were annotated using dfast [31], followed by manual curation. Draft genomes were also annotated using dfast. The complete genome sequences of strains 10942, 112648 and 122715, and the draft genome sequences of 99 O145:H28 strains obtained in this study were deposited in GenBank/EMBL/DDBJ under BioProject accession numbers starting from PRJDB8147 (see Table S1 for the individual accession numbers).

### SNP detection and phylogenetic analysis

The SNP sites (7255 sites) on the PP-/IE-/insertion sequence (IS)-free and recombination-free chromosome conserved backbone (3 572 740 bp) were identified using Gubbins [32] and MUMmer [33], and used for the reconstruction of the ML tree with RAxML [34] as previously described [29]. Clustering analysis was performed using the hierBAPS program with parameters of L=3 and maxK=5 [35]. ML trees were displayed using iTOL [36] or FigTree v.1.4.3 (http://tree.bio.ed.ac.uk/software/figtree/).

### Temporal analysis

Excluding 6 genomes lacking the temporal information, 233 genomes were analysed using 7029 SNP sites in a 3 572 740 bp recombination-free core backbone. The temporal signal in the ML trees was investigated using TempEst [37] to assess the linear relationship between the root-to-tip distance and the year of isolation. Using jModeltest v.2.1 [38], the TIM or GTR model of nucleotide substitution was selected as the best-fit model under both the Akaike and Bayesian information criteria. Further temporal analysis to date the important nodes was performed using beast v.1.8 [39]. The GTR substitution model with the relaxed lognormal clock and constant population size model was selected as the best-fit model by assessing the Bayes factor. Subsequent analyses were performed as previously described [29], and the results were summarized as a maximum clade credibility tree using TreeAnnotator in beast and visualized with FigTree v.1.4.3.

### Identification of PPs, IEs and IS elements

PPs and IEs in the complete genomes were searched for using the phaster web server (http://phaster.ca) [40], followed by manual curation to precisely define each element. Sequence similarities between PPs or IEs were analysed using blastn, and the results were visualized as a heatmap using R v.3.5.1. ISfinder [41] was used for IS searches with a threshold of >95 % identity and *E* value <1×10^−20^. If multiple matches to highly similar ISs (>95 % identity and >99 % coverage) were detected, the IS type was assigned to the best hit.

### Analyses of the plasmid replicons, integrase genes, pangenome and virulence-related genes

The presence of plasmid replicons was determined using PlasmidFinder v.1.3 [42] with default parameters. Genes annotated as integrase genes in all the O145:H28 genomes [except for truncated ones (<400 bp)] were clustered using cd-hit (threshold: >97 % identity and >40 % alignment coverage) [43] to determine the presence of each integrase gene cluster in each strain (see Table S2 for all integrase gene clusters). Integration sites of PPs/IEs associated with each integrase gene/gene cluster were determined according to the PP/IE annotation results when the integrase gene was present in either of the seven complete genomes. When the integrase gene was absent in any of the complete genomes, one draft sequence that contained the gene was selected and the relevant scaffold/contig was manually analysed to determine the integration site of the PP/IE associated with the integrase gene. To validate the accuracy of integration site prediction based on these data, we randomly selected two or more draft genomes for each integrase gene (except for those specific to one or two strains) and analysed the scaffold/contig that contained the integrase gene to examine whether the PP/IE associated with the integrase was actually present at the predicted integration site. The results of this validation analysis were concordant with the prediction of all integrase genes/gene clusters examined, indicating the accuracy of our prediction. All O145:H28 genomes were annotated by dfast, and the core and accessory genes were identified using Roary with default parameters except for the definition of core genes (genes present in 100 % strains) and the clustering option (don’t split paralogs) [44]. Plasmid- and chromosome-associated genes were identified using PlaScope [45] and blastp searching in an in-house phage-associated gene database (Table S3) as outlined in Fig. S1. Stx subtypes were determined by *in silico* PCR [https://github.com/bowhan/kent/tree/master/src/isPcr] with previously described primers [6] and default conditions, except for the requirement of a 10 bp perfect match at the 3′ end of the primer. The results of *in silico* PCR were confirmed by blastn search of each draft genome using the reference sequences of each subtype [6] with a threshold of >90 % identity and >60 % coverage. The result of *in silico* PCR was assigned as ‘confirmed’ when the top hit showed >99 % identity and >99 % coverage to the subtype determined by *in silico* PCR. When the identity of the top hit was lower than 99 % (31 cases), the results of blastn search were further inspected individually. In all of the 31 cases, the top hits matched those determined by *in silico* PCR (all were >98.9 % identity and 100 % coverage). Thus, this confirmation analysis completely confirmed the results of *in silico* PCR. To analyse T3SS effector and plasmid-associated virulence genes, all intact effector genes and plasmid virulence genes identified in strains 10942 and RM13516 were clustered using cd-hit with a threshold of >90 % identity and >30 % coverage. The representative sequences of each cluster and three effector genes that were absent in the seven complete genomes were used to create an in-house database (Table S2) for blastn searches (threshold:>90 % identity and >30 % coverage).

## Results

### Phylogenetic overview of O145:H28

**Fig. 1.**
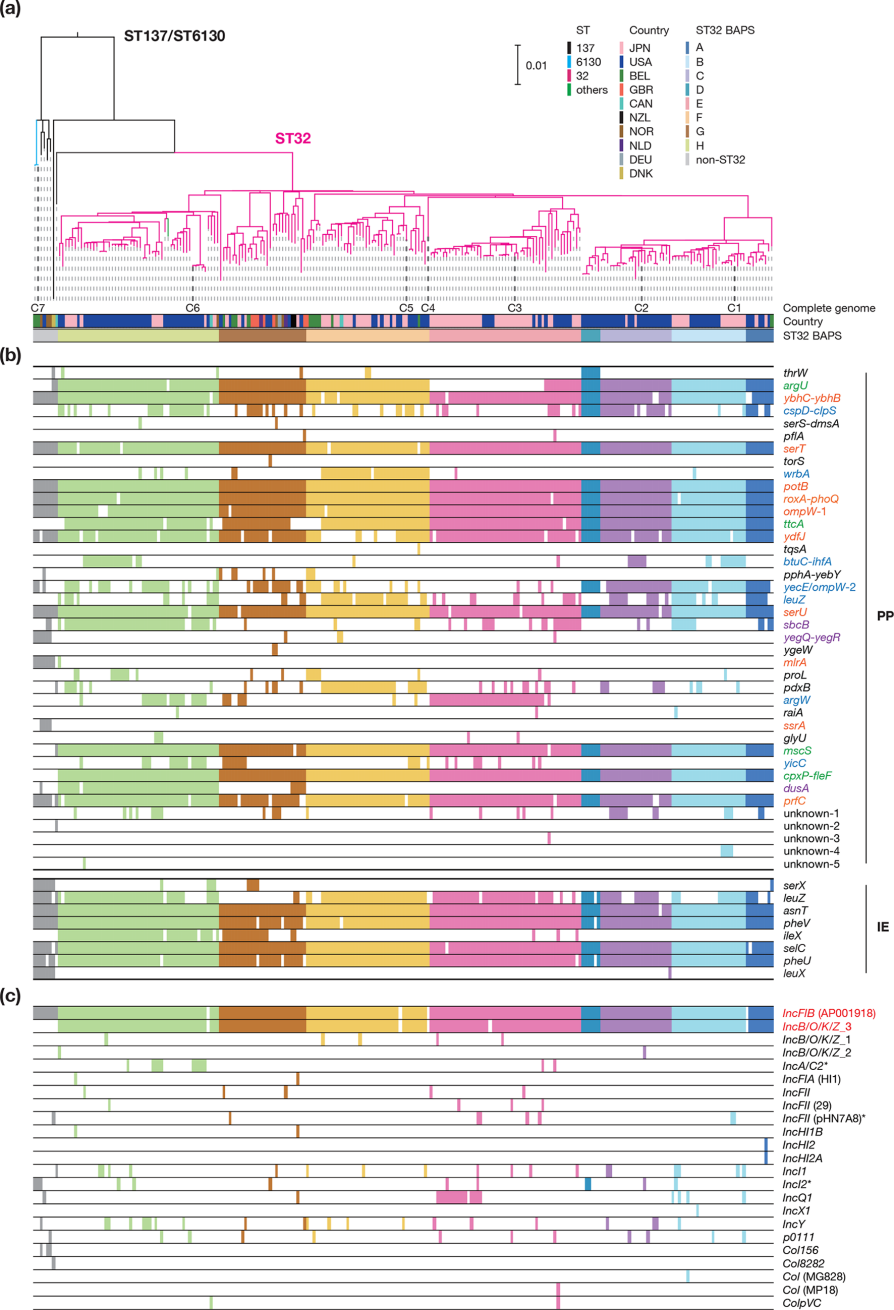
The phylogenetic relationship of the 239 O145:H28 STEC strains and their repertoires of integrase genes and plasmid replicons. (a) A ML tree of the 239 O145:H28 strains. The tree was reconstructed based on the recombination-free SNPs (7255 sites) identified on the chromosomal backbone sequence (3 564 334 bp) of the 239 strains and rooted by the O157:H7 strain Sakai. C1–C7 indicate the seven completely sequenced strains (C1, 10942; C2, RM9872; C3, 122715; C4, 95–3192; C5, 112648; C6, RM13514; C7, RM13516). Eight clades of ST32 identified by hierBAPS and geographical information of the strains are indicated. (b) The distribution of integrase genes. Integrase genes are represented by the integration sites of the PPs and IEs where each integrase gene is located. The four groups of integration sites (I–IV) defined based on the integration patterns in the seven complete genomes (see Fig. 2a) are indicated by the following colours: I, orange; II, green; III, blue; and IV, purple. (c) The distribution of plasmid replicons. Plasmid replicons were detected by PlasmidFinder. Two replicons of the Vir plasmid are indicated by red, and those of other plasmids found in the seven completely sequenced strains are indicated by asterisks. Coloured and open boxes in (b) and (c) indicate the presence or absence of each integrase gene or plasmid replicon, respectively. Bar, the mean number of nucleotide substitutions per site. JPN: Japan, USA: United States of America, BEL: Belgium, GBR: United Kingdom, CAN: Canada, NZL: New Zealand, NOR: Norway, NLD: Netherlands, DEU: Germany, DNK: Denmark.

We analysed the WGSs of a total of 239 strains isolated in 10 countries, which comprised the 99 genomes of Japanese and Belgian strains sequenced in this study and 140 publicly available genomes (Tables 1 and S1). While most strains belonged to sequence type (ST)32 (*n*=230), 6 and 2 strains belonged to ST137 and ST6130, respectively. ST32 and ST6130 are both single-locus variants of ST137. The WGS-based phylogeny of the 239 strains revealed that ST137 is ancestral to ST32 and ST6130 (Fig. 1a). ST32 strains were separated into eight distinct clades (A–H). A temporal analysis of O145:H28 by the Bayesian coalescent analysis [46] estimated that ST32 branched from the ST137 lineage in 1786 (95% highest posterior density: 1706–1846), and the separation of ST32 into multiple clades occurred during a relatively short period, approximately 130–150 years ago. Intriguingly, although Japanese and USA ST32 strains showed an unbiased distribution, most European strains belonged to ST137/ST6130 and the ST32 clade G, the latter of which was first separated from other ST32 clades (Fig. S2). This biased distribution may suggest the European origin of O145:H28 STEC, although additional European strains need to be analysed.

**Table 1.** The strain set analysed in this study

Country	Source						*stx* genotype					
	Human/clinical	Animals	Foods	Unknown	Total		*stx1a*	*stx1a*/*stx2a*	*stx1a*/*stx2d*	*stx2a*	*stx2c*	*stx2d*
Japan	88	0	0	0	88		26	13	1	47	1	0
USA	98	11	7	0	116		25	23	0	58	6	1
Belgium	10	0	3	0	13		3	0	0	9	1	0
UK	7	0	0	0	7		1	0	0	5	0	0
Other countries	11	2	0	2	15		4	0	0	7	0	0
Total	214	13	10	2	239		59	36	1	126	8	1

### General features of the complete O145:H28 genomes

We determined the complete genome sequences of three ST32 strains in our strain set (strains 10942, 122715 and 112648), which were genetically divergent from the four previously sequenced strains (strains RM9872, 95–3192, RM13514 and RM13516) (Table 2, Fig. 1a) [25, 47, 48]. The chromosomes of the seven strains (referred to as C1 to C7; C7 belonged to ST6130, while the others belonged to ST32) were 5374–5585 kb in size and contained various numbers of PPs (12–20) and IEs (6 or 7). These strains, except for C3, carried one or two large plasmids (59–98 kb). The chromosome backbones of the seven strains were well conserved and exhibited overall genomic synteny, excluding small inversions in several strains (Fig. S3a). A 275 kb region was unique to the C7 genome compared to the ST32 genomes, and contained two deletions, an inversion, unique IE insertions and internal segments showing only 97–98 % sequence identity to the ST32 strains (Fig. S3b). These features suggest that this segment was acquired from other *E. coli* lineages, because the mean sequence identities between the seven O145:H28 were >99.57 %, while those between various *E. coli* strains are variable but >95.7 % [49]. Similarly, the IS repertoire of C7 differed significantly from that of the ST32 strains (Table S4). This difference appears to reflect the independent evolution of the ST32 and ST137/ST6130 lineages.

**Table 2.** General genomic features of the seven completely sequenced O145:H28 STEC strains

Feature	ST (clade)						
ST32 (B)	ST32 (C)	ST32 (E)	ST32 (F)	ST32 (F)	ST32 (H)	ST6130
Strain	10942	RM9872*	122715	95–3192†‡	112648	RM13514*	RM13516*
Strain ID in this paper	C1	C2	C3	C4	C5	C6	C7
Accession no.	AP019703-5	CP028379-80	AP019708-10	CP027362	AP019706-7	CP006027-9	CP006262-4
Reference	This study	Parker *et al.* [47]	This study	Patel *et al.* [48]	This study	Cooper *et al.* [25]	Cooper *et al.* [25]
Chromosome (kb)	5374	5385	5418	5385	5488	5585	5402
CDSs	5090	5114	5121	5143	5229	5613	5324
rRNA operons	7	7	7	7	7	7	7
tRNAs	100	106	103	102	104	104	98
PPs	17	16	16	16	18	20§	12§
IEs	6	6	6	6	6	7§	7§
IS elements	62	70	66	61	60	72§	66§
Plasmid (kb)	92/71	89	87/49		91	87/65	98/59
CDSs (plasmid total)	176	92	152		87	163	188
IS elements	26	23	23		24	28§	16§
Total genome size (kb)	5537	5474	5554	5385	5579	5737	5559

CDS, protein-coding sequence.

*Re-annotated using the same pipeline in this study.

†Annotated in this study.

‡No plasmid sequences have been deposited.

§Re-identified using the same pipeline in this study.

### Variations in PPs and IEs

#### PPs and IEs in the complete genomes

**Fig. 2.**
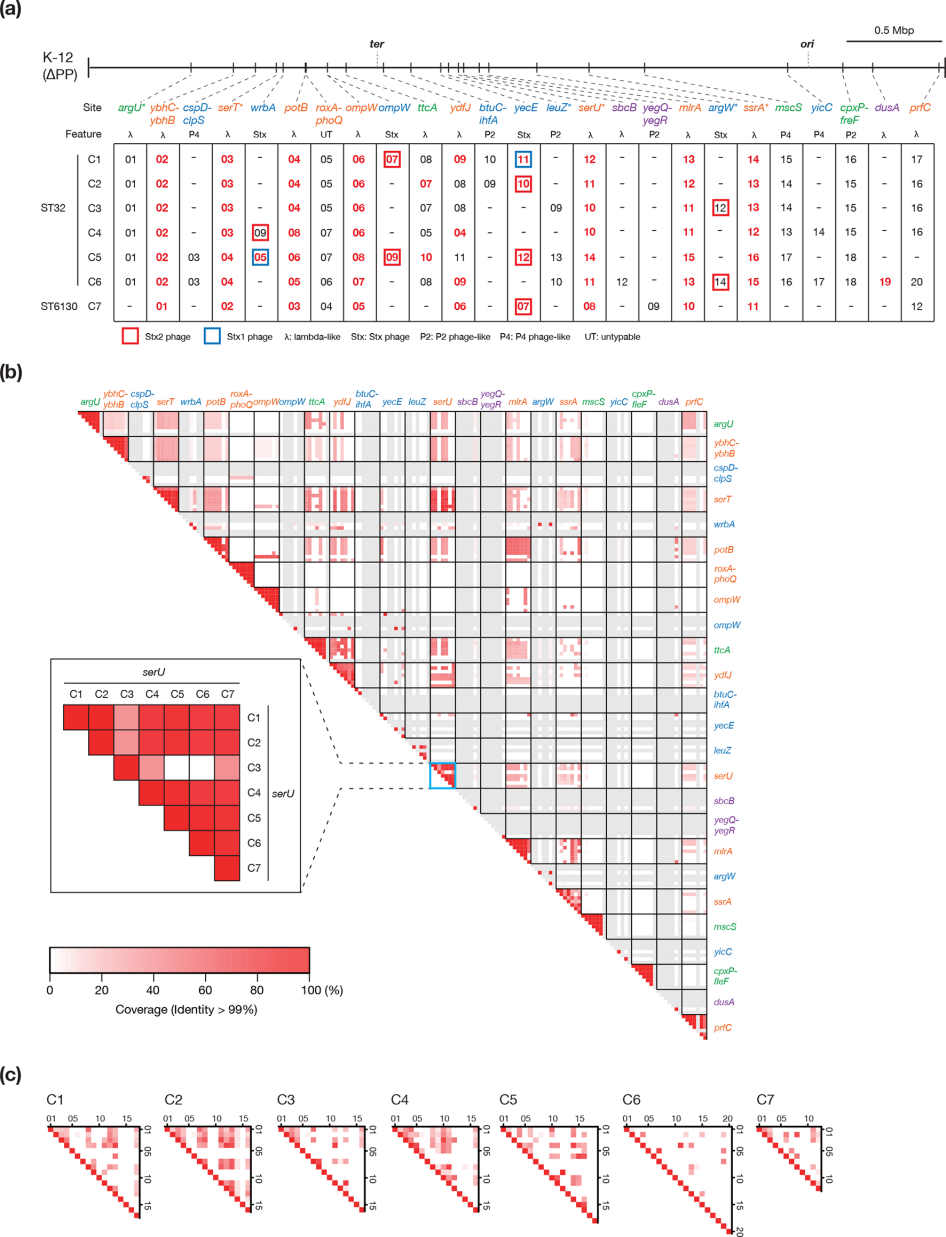
Conservation and variation of the PPs in the seven completely sequenced O145:H28 genomes. (a) The chromosomal integration sites of the PPs identified in seven complete genomes are shown on the PP-removed chromosome backbone of strain K-12 MG1655 (K-12 ∆PP). Integration sites are indicated by the name of the gene (or intergenic region) in which each PP is integrated. Based on the integration patterns, integration sites are classified into four groups: (I) integrated into all strains (orange), (II) absent in only one strain (green), (III) integrated into two to four strains (blue), and (IV) integrated into only one strain (purple). T3SS effector-encoding PPs are indicated in red and bold. tRNA and transfer-messenger RNA (tmRNA) genes are indicated by asterisks. (b) The results of all-to-all nucleotide sequence comparison of the PPs identified in the seven complete genomes. PPs were grouped according to their integration sites. Coloured boxes indicate each pair of PPs compared. Alignment coverage between two PPs (the percentage of the longer sequence) with >99 % nucleotide sequence identity is indicated by a heatmap. In the inset, an enlarged view of the comparison of PPs integrated in the *serU* locus is shown as an example. Empty sites (no PP integration) in each strain are indicated by grey boxes. (c) The results of all-to-all nucleotide sequence comparison of the PPs within each genome. The alignment coverage between the two PPs compared is shown in (b).

We identified a total of 25 integration sites for PPs. These sites were classified into four groups (Fig. 2a): (I) for nine sites, PPs were present in all strains (common sites); (II) for five sites, PPs were present in all but one strain; (III) for eight sites, PPs were present in two to four strains (variable sites); and (IV) for three sites, PPs were found in only one strain (strain-specific sites). At four of the five group II sites, PPs were integrated into only the ST32 strains (ST32-specific sites). At the remaining group II site, PP was absent in only C5, suggesting strain-specific PP deletion. Thus, we regarded this site as a common integration site, and the numbers of group I and II sites were 10 and 4, respectively. Most PPs in group I and II sites (11/14) were lambdoid phages, reflecting the fact that lambda-like phages dominate the PP pool of each strain, as in other STECs [9, 50]. Notably, the integration sites of Stx phages were highly variable (four sites identified), suggesting that Stx phages have relatively recently been acquired in a clade- or strain-specific manner.

All-to-all comparison of the PPs based on the genomic coverage of sequences that shared more than 99 % identity (Fig. 2b) revealed that the genomes of PPs at the group I and II sites were relatively well conserved, although replacements and deletions of various lengths have occurred in many cases, particularly at *potB*, *ydfJ*, *serU*, *mlrA* and *ssrA*. Because a substantial amount of shared sequences was observed among the lambdoid phages in each strain (Fig. 2c, Table S5) and among those integrated in different loci in different strains (Fig. 2b), both recombination with incoming phages and inter-PP recombination in host strains appear to have contributed to PP diversification. Group III and IV sites (*n*=11) indicate frequent acquisition of PPs, although PP deletion may have occurred at some group III sites. Taken together, this analysis revealed the dynamic changes in the PP genomes acquired by the common ancestors of the O145:H28 or ST32 lineage and the frequent acquisition of PPs.

**Fig. 3.**
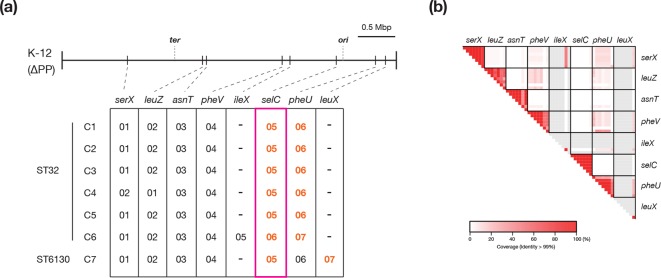
Conservation of and variation in the IEs. (a) The conservation of and variation in the IEs in the seven completely sequenced O145:H28 genomes are shown. The integration sites of the identified IEs are shown on the PP-removed chromosome backbone of strain K-12 MG1655 (K-12 ∆PP). Integration sites are indicated by the names of genes in which each IE is integrated. Note that all eight genes are tRNA genes. The LEE (in the *selC* locus) is indicated by a magenta rectangle. T3SS effector-encoding IEs are indicated in orange and bold. (b) The results of all-to-all nucleotide sequence comparison of the IEs identified in the seven complete genomes are shown. IEs were grouped according to their integration sites. Similar to Fig. 2, coloured boxes indicate each pair of IEs compared, and alignment coverage between two IEs (the percentage of the longer sequence) with >99 % nucleotide sequence identity is indicated as a heatmap. Empty sites (no PP integration) in each strain are indicated by grey boxes.

In contrast, IEs were highly conserved (Fig. 3; IEs were defined as genomic segments containing an integrase gene and signs of integrase-mediated integration, but no genes for apparently phage-related functions [9]). We identified six or seven IEs in each strain and eight integration sites in total. At two sites, IEs were found in one strain (strain specific); however, the genomes of the IEs integrated in the remaining six sites were well conserved, even in the ST6130 strain (C7). The C6-specific IE (at *ileX*) was generated by the duplication of the IE at *serX*, and the C7-specific IE (at *leuX*) was generated by the inversion between the IE at *pheU* and a novel IE (Fig. S3b).

#### PPs and IEs in the entire O145:H28 lineage

Due to the highly fragmented features of the PP and IE sequences in draft sequences, the same level of detailed analysis applied to the complete genomes was difficult to apply to the draft sequences. Therefore, we estimated the variations in PPs and IEs on the basis of integrase gene repertoires (Fig. 1b). This analysis revealed that the *int* genes of the PPs at group I and II integration sites were well conserved in the strain set (Fig. 1b). Three genes responsible for the integration at three group I and II sites (*argU*, *mlrA* and *ssrA*) showed poor conservation; however, these genes were deleted from the PPs in strain C3 (*argU*) or all complete ST32 genomes (*mlrA*, *ssrA*). Thus, similar deletions of *int* probably occurred in the ST32 clade E (including C3) or the entire ST32 lineage. In contrast, most *int* genes of the PPs at group III and IV sites showed clade-specific or highly variable distributions, suggesting the clade-specific or sporadic acquisition of these PPs. Sixteen *int* genes that were not present in the seven complete genomes were detected in very few strains. These results support our hypothesis that while the integrations of PPs at group I and II sites were ancestral events, the acquisition of PPs at other sites has frequently occurred in O145:H28.

Among the eight IE-associated *int* genes identified in the complete genomes, the gene was deleted from the IEs at *serX* in all ST32 strains. Thus, this gene was not detected in most of the ST32 strains. However, those of the five IEs found in all complete genomes were well conserved in the entire O145:H28 population, except for the genes of the IEs at *leuZ* and *ileX*. The former was not detected in various strains, especially the ST32 clade G strains, while the latter was found in the ST32 clades G and H and an additional few ST32 strains. The gene of the IE at *leuX* was specific to ST137/ST6130. These results suggest that the six IEs were acquired by a common ancestor of O145:H28, and five of these IEs, including the IE corresponding to the LEE (at *selC*), have been stably maintained.

### Variations in plasmids

#### Plasmids in the complete genomes

The Vir plasmids of the ST32 strains were nearly identical except for several small structural variations, most of which were associated with IS elements (Fig. S4a). Notably, these ST32 Vir plasmids were very similar to those of STEC O26:H11, suggesting plasmid transmission between O145:H28 ST32 and O26:H11 (Fig. S4b). In contrast, the Vir plasmid of C7 shared limited parts of the genome with the ST32 plasmids, indicating large genome replacement of the Vir plasmid in either lineage. One additional plasmid (NV plasmid) was present in four strains, but they were distinct from each other except for several small regions shared by the C1 and C6 NV plasmids (Fig. S4c). The C1 plasmid was a conjugative and multidrug resistance plasmid, and the C3 plasmid had a plasmid PP-like structure (Fig. S4d) [51]. As reported elsewhere [25], the C6 plasmid carried multiple resistance genes, and the C7 plasmid was characterized by gene clusters for type IVb pilus biosynthesis and a type IV secretion system.

#### Distributions of plasmid replicons in the entire O145:H28 lineage

As plasmids are also often highly fragmented in draft genomes, we analysed the repertoires of plasmid replicons using PlasmidFinder [42] (Fig. 1c) and identified many plasmid replicons in the strain set. Notably, the Vir plasmids, which contain *IncFIB* and *IncB/O/K/Z*_3 (the ST32 plasmids) or only *IncFIB* (the ST6130 plasmid), were highly conserved in the entire O145:H28 population. In contrast, all other plasmids (21 types of replicons in total) exhibited a highly variable distribution, and each was present in few strains. This result indicated that while the Vir plasmids have been stably maintained, the frequent gain and loss of various plasmids have occurred across the O145:H28 lineage.

### Impacts of PP and plasmid variations on the pangenome structure

**Fig. 4.**
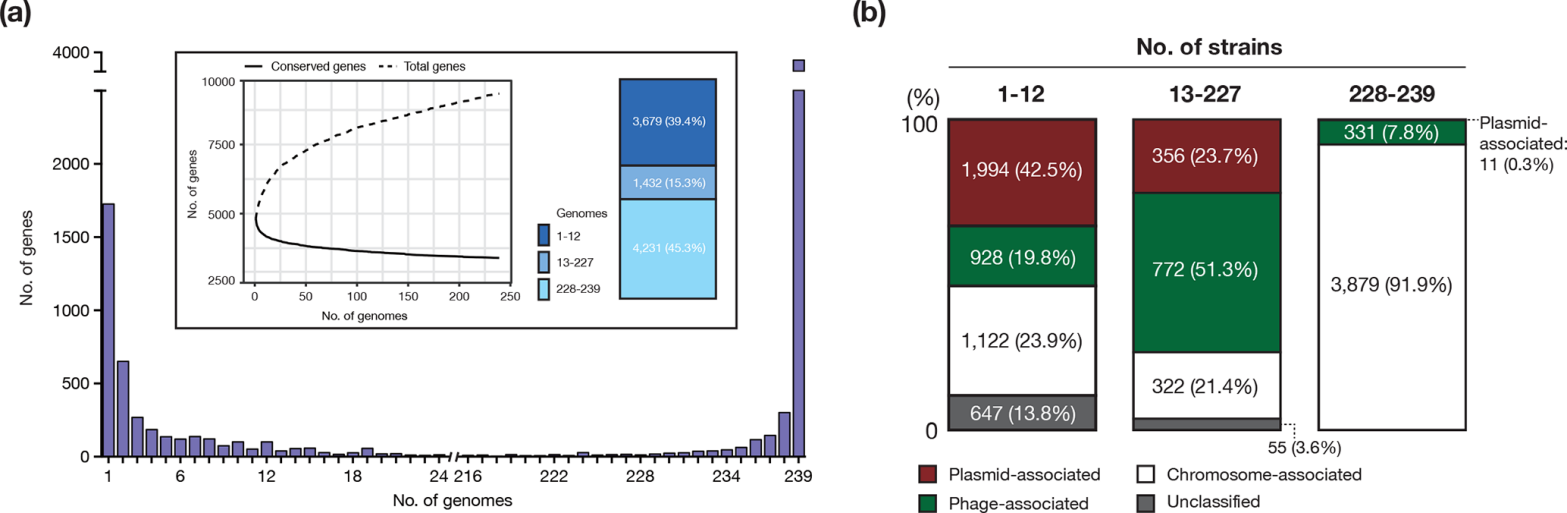
Plasmid- and phage-associated genes in the pangenome of O145:H28. (a) Genes were analysed by frequency histogram (the number of genes present in any given number of genomes indicated on the *x*-axis). The pangenome (total genes; dashed line) and the core genome (conserved genes shared by all strains; solid line) profile curves are shown in the inset. The proportion of three groups of genes, which were grouped according to the frequency in the 239 genomes, is also shown. (b) The ratios of plasmid- and phage-associated genes in three groups of genes (the rarely present, variable present and conserved genes) are shown. The three groups were classified according to the frequency in the 239 strains. The plasmid-, phage- and chromosome-associated genes and unclassified genes were identified by a stepwise classification (see the main text).

As the marked variation in PPs and plasmids became evident, we investigated the effects of this variation on the pangenome of O145:H28. The identified pangenome comprised 9342 genes, representing an ‘open’ pangenome (Fig. 4a). Large numbers of rarely present genes (3679 genes; present in less than 5 % of the strains) and variably present genes (1432 genes; present in 5~95 % of the strains) were identified. To estimate the ratios of plasmid- and phage-associated genes in each category, we classified all genes of the 239 strains into three classes, namely, plasmid-associated, chromosome-associated and unclassified by the stepwise classification outlined in Fig. S1. This analysis revealed that plasmid- or phage-associated genes occupied 62.3 % of the rare genes and 72.2 % of the variably present genes (Fig. 4b). A clear difference between the rare genes and the variably present genes was also evident; plasmid-associated genes were highly abundant among the rare genes (42.5 %), while phage-associated genes were highly abundant among the variably present genes (51.3 %). The core genome (*n*=3804) contained a considerable number of phage-associated genes (331 genes), but only 11 plasmid-associated genes (all were on the Vir plasmids). These results are consistent with the abovementioned findings of the relatively stable maintenance of a set of PPs acquired by the common ancestor of O145:H28 and the frequent acquisition (and loss) of various PPs and plasmids.

### Variations in major virulence-related genes

**Fig. 5.**
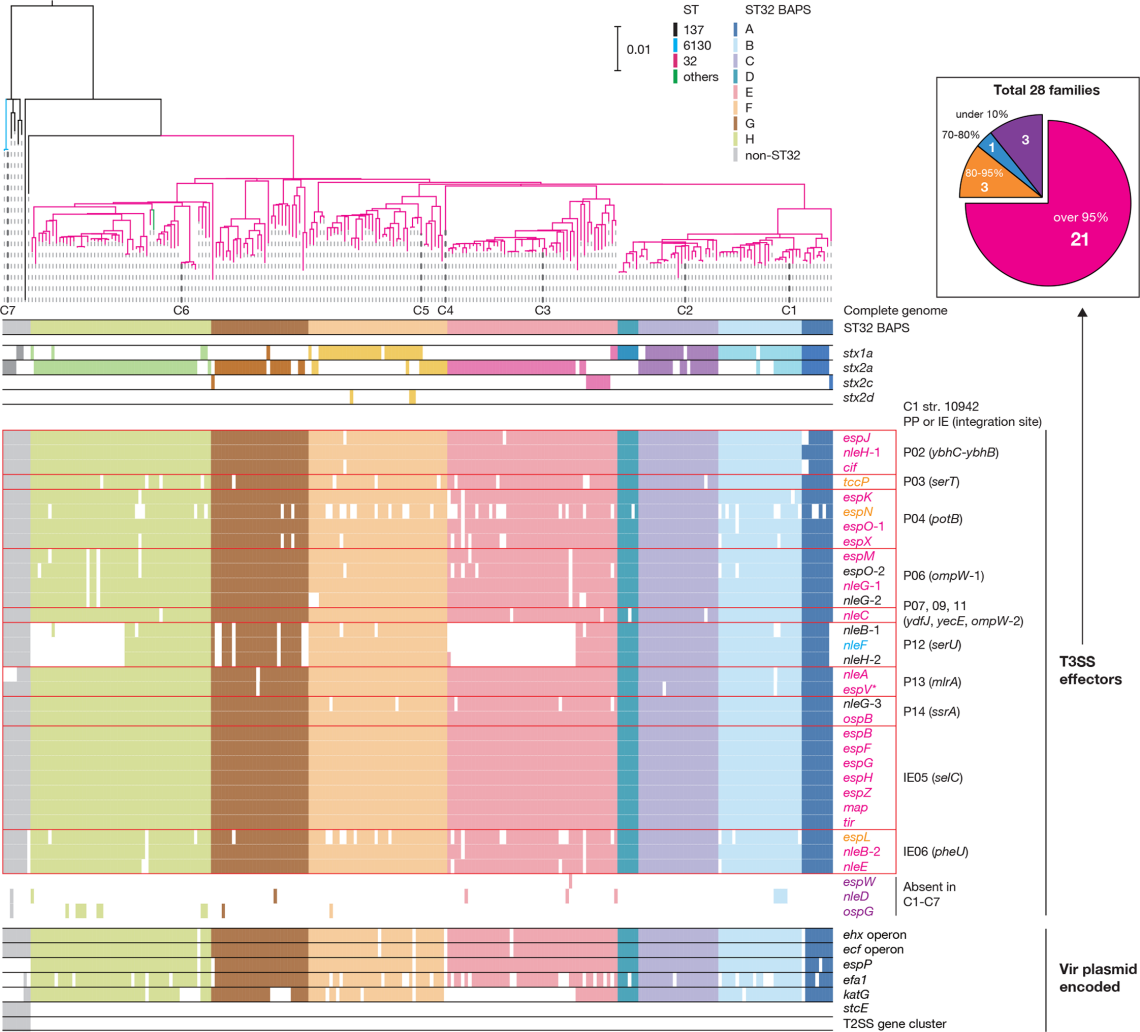
The repertoires of major virulence-related genes in the 239 O145:H28 strains. Along with a ML tree of the 239 O145:H28 strains (the same tree shown in Fig. 1), the presence or absence of each subtype of *stx* (*stx1a*, *stx2a*, *stx2c* or *stx2d*), PP- and IE-encoded T3SS effector genes, and Vir plasmid-encoded virulence genes in each strain is shown. The PPs and IEs of strain 10942 (C1) that contain each T3SS effector gene are indicated. Three T3SS effector genes (*nleD*, *espW* and *ospG*) were absent in the seven complete genomes. Coloured and open boxes indicate the presence or absence of each gene/operon/gene cluster. In the inset, 28 effector families identified in the 239 strains have been classified into four groups according to the numbers of genomes where each family is conserved, and the numbers of effector families in each group are shown. The colours of effector genes in the main figure indicate the effector family groups of each effector (the same colours as used in the inset). Bar, the mean number of nucleotide substitutions per site. The reference sequence of the *espV* gene (indicated by an asterisk) was taken from C7 strain RM13516 (see Methods).

As many virulence-related genes are associated with PPs and plasmids, we finally investigated the variation in virulence gene repertoires in O145:H28 with a particular focus on three major groups of VFs in STEC: Stxs, T3SS effectors and plasmid-associated VFs (Fig. 5).

### Stxs

We identified four *stx* genes encoding Stx1a, Stx2a, Stx2c and Stx2d. These genes showed a highly variable distribution; although *stx2a* was relatively widely distributed, the distribution of *stx1a* was biased toward five ST32 clades (A–D, F). The *stx2c* and *stx2d* genes were present in a few ST32 strains. The variation in *stx* distribution is apparently underrepresented in this analysis, because the integration sites of Stx phages in the complete genomes were highly variable even between the phages encoding the same Stx subtype (Fig. 2a) and strain C5 contained two Stx2a phages. Therefore, the history of the acquisition of and alteration (exchange or loss) in Stx phages in O145:H28 is complex. To comprehensively understand this history, analysis of the Stx phages from additional O145:H28 strains is required.

### T3SS effectors

In the complete genomes, we identified effector genes for 28 effector families (34–40 copies per genome). The effector repertoires of the complete genomes were similar but with some variations in the copy numbers of several effector families (Table S6) and an exceptional absence of the *nleB*-1, *nleF* and *nleH*-1 genes in C3, which was caused by a large genomic replacement in the PP at *serU*. All effectors were encoded by PPs or IEs, as observed in other STEC lineages [8, 9]. Notably, 8 of the 10 PPs integrated into the group I sites encoded effectors (Fig. 2a).

Analysis of the entire O145:H28 lineage revealed the high conservation of effector genes identified in the complete genomes (Fig. 5). The exceptions were the loss of *nleB*-1, *nleF* and *nleH*-1 in two sublineages in the ST32 clades E and G, and the relatively frequent loss of several effectors, such as *espN* and *espL*. The former finding was consistent with the genome replacement in the PP at *serU* of strain C3 (clade E). Similar genome replacements of PPs have probably occurred in the sublineage of clade G. Three effector families (*espW*, *nleD* and *ospG*) that were absent in the complete genomes were identified, but these effector genes were found in a few strains. These findings indicated that most effector genes found in the seven complete genomes were acquired by the common ancestor of O145:H28 through the acquisition of multiple PPs and IEs, and were stably maintained despite the dynamic changes in PP pools. Importantly, this analysis identified 21 effector families conserved in more than 95 % strains, which appear to represent the ‘core T3SS effectors’ of O145:H28.

### Plasmid-encoded VFs

Consistent with the variation in Vir plasmids observed in the complete genomes, only two of the seven virulence genes/operons (the *ehx* and *ecf* operons) were well conserved in the entire O145:H28 population (Fig. 5). Among the other three genes on the ST32 Vir plasmids, *espP* was highly conserved, but *efa1* and *katG* showed frequent loss in the ST32 lineage. Two virulence genes/gene clusters found in only the ST6130 Vir plasmid were specific to ST137/ST6130. Interestingly, the *efa1* and *katG* genes were detected in the two ST137 strains (only *efa1* in one strain) that were most closely related to ST32. This finding suggests that these two strains may contain hybrid Vir plasmids representing a transient form that evolves into the ST32 Vir plasmid.

## Discussion

Our analysis of multiple complete genomes and a large draft genome set of O145:H28 provided a global phylogenetic overview of this STEC lineage and revealed the dynamics of PPs and plasmids during the diversification of O145:H28. Among the three STs identified, ST137 is the ancestral lineage, and ST32 is the currently circulating major lineage. ST32 branched from ST137 approximately 230 years ago. It has been shown that the major lineages of O157:H7 and O26:H11 also emerged approximately 170 and 213 years ago, respectively [29, 52]. Thus, the common ancestors of the currently circulating major clones of these three STEC lineages emerged during the nearly same era, approximately 200 years ago. This finding may suggest some common environmental or social factors exerted on the generation and selection of these clones.

Comparison of the seven complete genomes that represent most of the major sublineages revealed the different dynamics of PPs, IEs and plasmids. This finding was confirmed by subsequent analyses of a large draft genome set using marker genes of each type of MGE. The analysis of the draft genome set further revealed different impacts of PPs and plasmids on the pangenome of O145:H28. Overall, PPs are more stably maintained than plasmids. In particular, PPs that were probably acquired by the common ancestor of O145:H28 (PPs at group I sites) and those acquired by the common ancestor of ST32 (PPs at group II sites) were well maintained in the entire O145:H28 and ST32 lineages, respectively, although various sizes of genomic deletion and replacement have occurred in each PP. Acquisition of various PPs has also occurred during the diversification of O145:H28. Importantly, Stx phages are among such PPs, and Stx phages have relatively recently been acquired by multiple clades or sublineages. Such dynamics of PPs resulted in the high proportion of phage-associated genes in the variably present genes in the pangenome. Although most IEs were acquired by the common ancestor of O145:H28 and have been stably maintained, the dynamics of plasmids clearly differs from those of PPs and IEs. The Vir plasmids are well conserved (at least in ST32), but the acquisition of a wide range of plasmids has occurred across the O145:H28 lineage. As the current plasmid repertoire of O145:H28 strains is a snapshot of changing repertoires, plasmid loss has also frequently occurred. Such dynamics of plasmids have had a strong impact on the pangenome, resulting in a high proportion of plasmid-associated genes in the rare gene fraction. Although we cannot exclude the possibility that some of the rare genes were artefacts generated by low sequence quality or some problems in genome assembling, the number of such artefacts was probably small because we excluded low-quality genome sequences from the data set and performed clustering analysis using Roary with the clustering option (don’t split paralogs).

The dynamics of PPs, IEs and plasmids have also had strong impacts on the virulence gene repertoires. The relatively recent acquisition of Stx phages in multiple clades or sublineages is directly linked to a highly variable distribution of the *stx* genes, although the composition of our strain set, which was heavily biased to human isolates, may have a potential influence on the observed distribution of *stx* genes. In contrast, many of the ancestrally acquired PPs (eight of the ten PPs at group I sites) encoded T3SS effectors were well conserved in the entire O145:H28 lineage, leading to the identification of 21 effector families that probably represent the core effectors of O145:H28. Although the roles of the LEE-encoded T3SS and its effectors in adaptation and survival in nature are not well understood, these factors may play important roles in not only pathogenesis in the human intestine but also adaptation and survival in other environments, such as the bovine intestine. The high conservation of the *ehx* and *ecf* operons on the Vir plasmid also suggests the important roles of these genes in the biology of O145:H28. These operons are also conserved in Vir plasmids of other STEC serotypes [9, 16, 53, 54], providing further support for this hypothesis. Increased attention must be focused on the roles and functions of these genes in adaptation and survival in various environments.

In conclusion, the systematic analysis of seven complete genomes and a large set of draft genomes has provided deeper insights into the dynamics and roles of MGEs in the evolution and diversification of STEC O145:H28, and shows the application of genomic analyses using both complete genomes and large draft genome sets. It is important, however, to select proper strains for the analysis of complete genomes to appropriately represent the entire population of the strains of interest.

## Data bibliography

1. Carrillo, C. BioProject ID: PRJNA319494 (2017).

2. Patel, P. BioProject ID: PRJNA218110 (2014).

3. Lewis, J.L. *et al.* BioProject ID: PRJNA72253 (2011).

4. Timme, R. BioProject ID: PRJNA218647 (2011), PRJNA218651 (2013).

5. Gangiredla,J. *et al.* BioProject ID: PRJNA230969 (2015).

6. Tillman, G. *et al.* BioProject ID: PRJNA268206 (2014).

7. Browne, A.S. BioProject ID: PRJNA414662 (2017).

8. Holmes A. BioProject ID: PRJNA419720 (2017).

9. Jostein, J., Kjersti, H. BioProject ID: PRJEB6447 (2014).

10. Gabrielsen, C. *et al.* BioProject ID: PRJNA275276 (2015).

11. Public Health England BioProject ID: PRJNA315192 (2018).

12. Statens Serum Institut. BioProject ID: PRJEB10700 (2015).

13. Mercer, R.G. BioProject ID: PRJNA277539 (2015).

14. Ferdous, M. BioProject ID: PRJNA285020 (2015).

15. Gao, X. BioProject ID: PRJNA445506 (2018).

16. Cooper, K.K. *et al.* BioProject ID: PRJNA178648-50 (2014).

17. Parker, C. BioProject ID: PRJNA69939 (2018).

## Supplementary Data

Supplementary material 1

Supplementary material 2
