# Supplementary material for: Differential dynamics and impacts of prophages and plasmids on the pangenome and virulence factor repertoires of Shiga toxin-producing Escherichia coli O145:H28
Source: Microb Genom. 2020 Jan 14;6(1):e000323. doi: 10.1099/mgen.0.000323 (PMC7067040; doi:10.1099/mgen.0.000323)
Supplement: Supplementary material 1 [file mgen-6-323-s001.pdf]

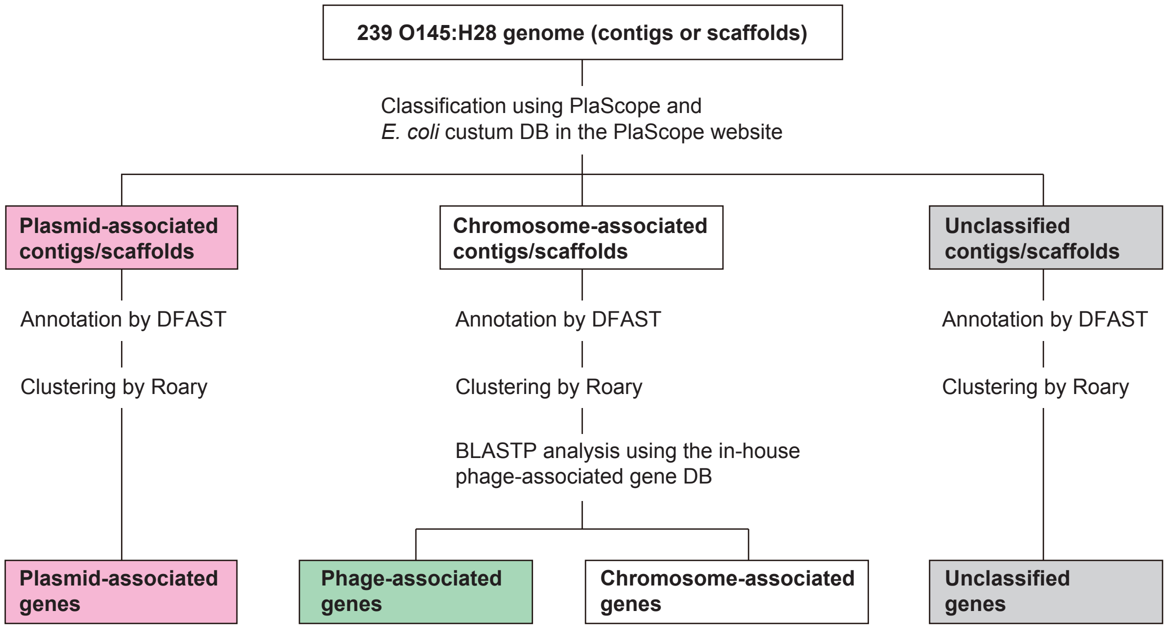

Fig S1. Stepwise classification of the O145:H28 genes.
To identify plasmid-associated genes, plasmid- and chromosome-associated scaffolds were extracted from each assembly using PlaScope with the *E. coli* custom database (DB) in the PlaScope website. Genes on the scaffolds classified as plasmid-associated, chromosome-associated, or unclassified were clustered using Roary to determine the proportion of plasmid-associated genes. The proportion of phage-associated genes in the chromosome-associated gene clusters was calculated using an in-house phage-associated gene DB, which was constructed in this study. This DB contained (i) all phage-associated genes identified in this study, (ii) genes identified by our previous genome sequencing of STEC O157:H7, O26:H11, O111:H-, and O103:H2; enteropathogenic *E. coli* (EPEC) O111:H- and O127:H6; and *Serratia marcescens* [1-5], and (iii) the publicly available phage genome data set reported previously [6] (see Table S3 for the details). The longest amino acid sequence in each gene cluster was used to perform a BLASTP search against the phage-associated gene DB constructed to identify the phage-associated gene clusters with a threshold of >60% identity and >60% alignment coverage.

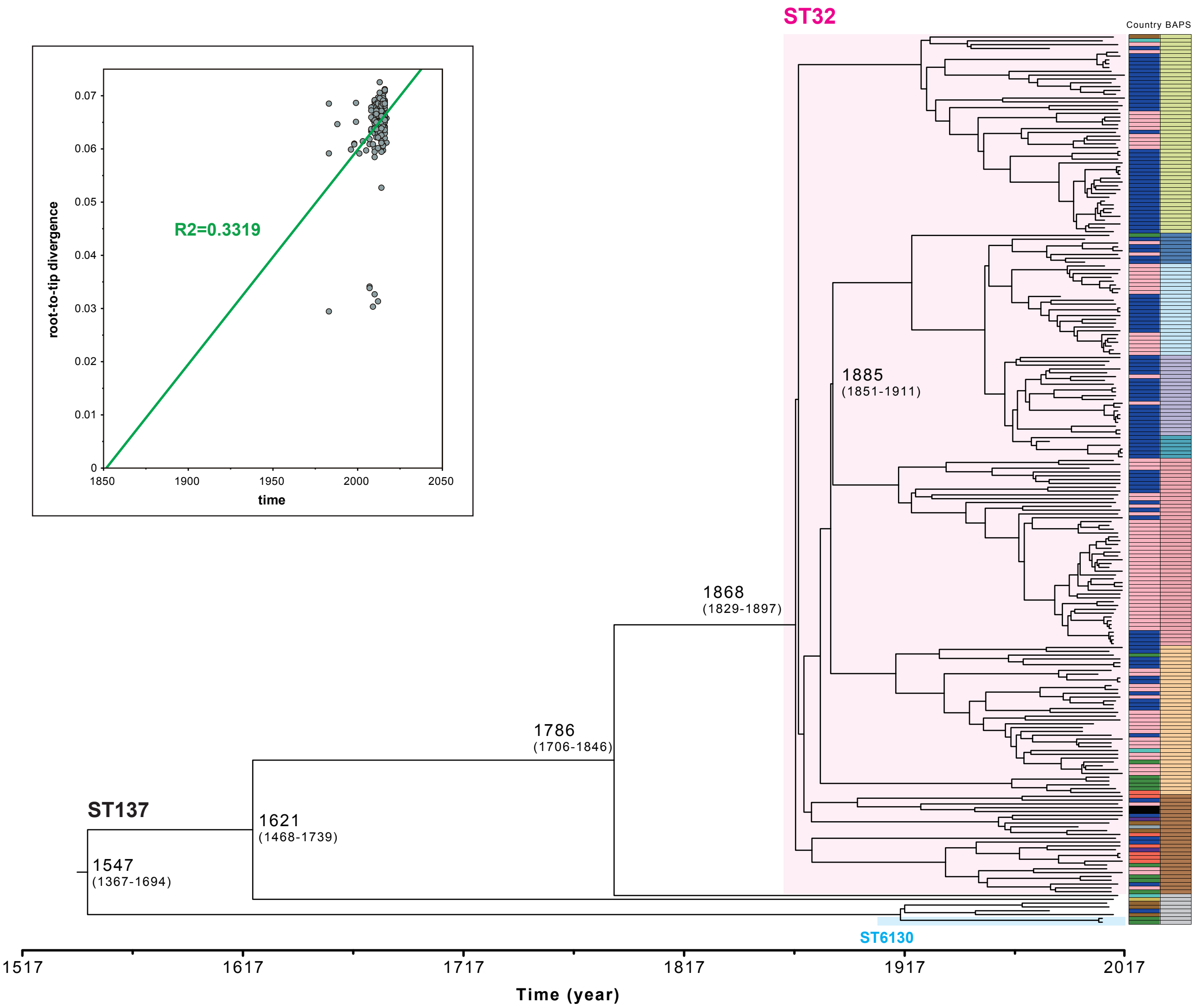

Fig S2. A maximum clade credibility tree representing the evolutionary history of O145:H28 strains inferred by Bayesian evolutionary analysis.

The result of regression analysis of root-to-tip distance against sampling date is shown in the inset, indicating positive correlation of genetic distance and sampling date. The time-calibrated phylogenetic tree was reconstructed using BEAST based on 5,208 concatenated recombination-free SNPs. The time to the most recent common ancestor (TMRCA) of each O145:H28 lineage is shown in the tree, and 95% highest posterior density (HPD) is indicated in parentheses. The BAPS clade and geographical information of each strain are also shown. Note that the overall topology of the tree was consistent with that of the ML tree shown in Fig. 1A in the main text.

A

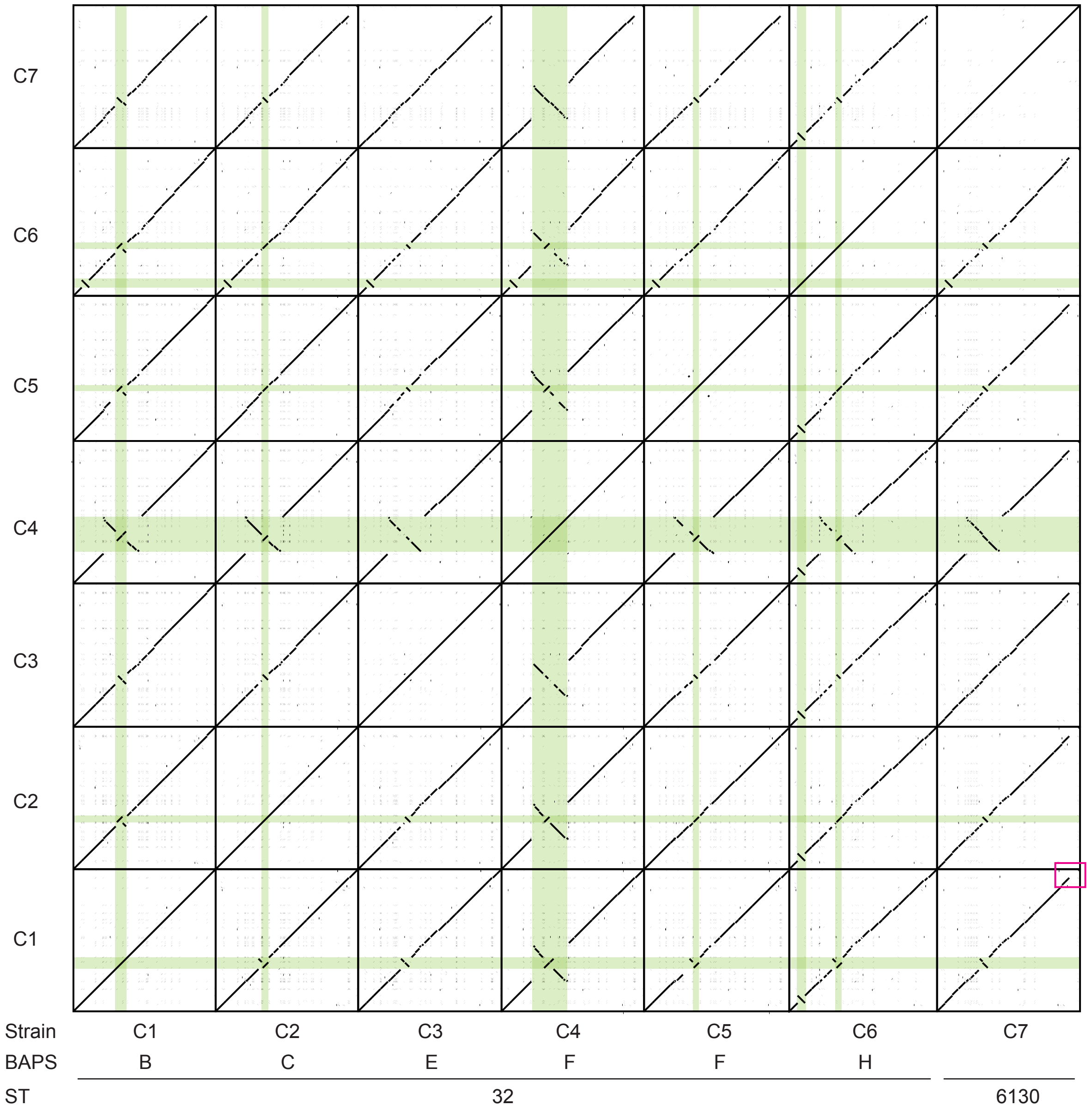

B

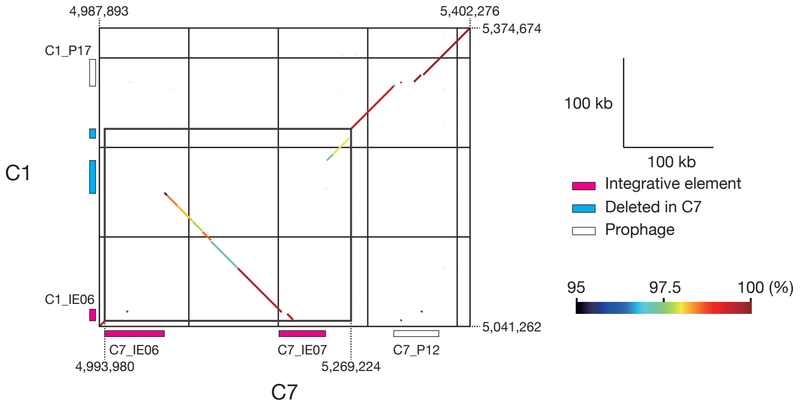

Fig S3. Comparison of the chromosome sequences of seven completely sequenced O145:H28 strains.
(A) Dot plot presentation of the conservation of chromosome sequences between the seven completely sequenced O145:H28 strains. BLASTN analysis (>99% sequence identity) was performed using the GenomeMatcher software [7] and results were displayed by the same software. The inversions identified are indicated by green shading. (B) An enlarged view of the chromosomal region indicated by a red rectangle in panel A. Sequence identities are indicated by heatmap.

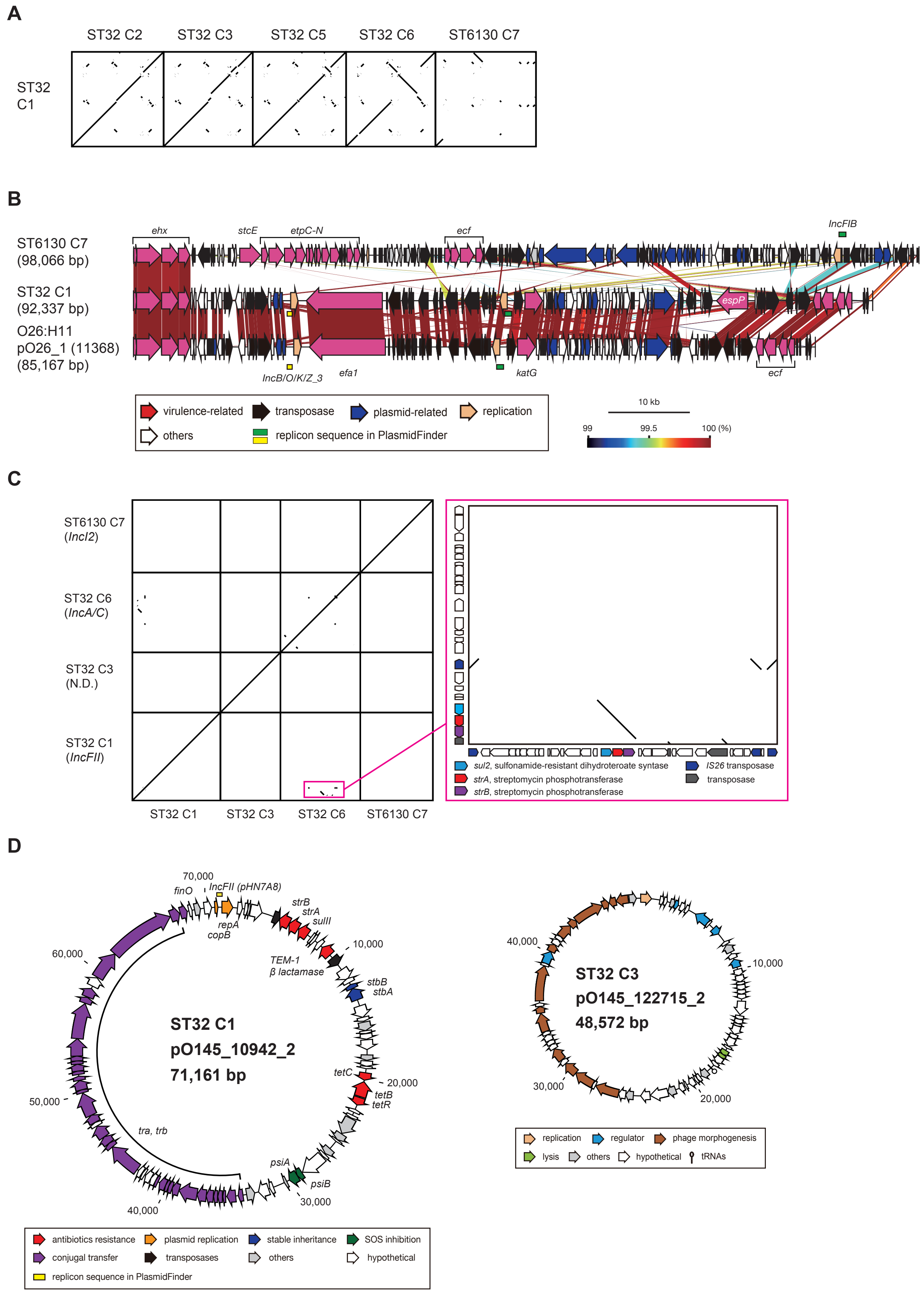
A
ST32 C2
ST32 C3
ST32 C5
ST32 C6
ST6130 C7
ST32 C1
B
ST6130 C7 (98,066 bp)
ST32 C1 (92,337 bp)
O26:H11 pO26_1 (11368) (85,167 bp)
ehx
stcE
etpC-N
ecf
IncFIB
espP
IncB/O/K/Z_3
efa1
katG
ecf
virulence-related
transposase
plasmid-related
replication
others
replicon sequence in PlasmidFinder
10 kb
99
99.5
100 (%)
C
ST6130 C7 (IncI2)
ST32 C6 (IncA/C)
ST32 C3 (N.D.)
ST32 C1 (IncFII)
ST32 C1
ST32 C3
ST32 C6
ST6130 C7
sul2, sulfonamide-resistant dihydroterоate syntase
strA, streptomycin phosphotransferase
strB, streptomycin phosphotransferase
IS26 transposase
transposase
D
ST32 C1
pO145_10942_2
71,161 bp
70,000
finO
IncFII (pHN7A8)
strB
strA
sulII
repA
copB
10,000
TEM-1 β lactamase
stbB
stbA
60,000
tetC
20,000
tetB
tetR
50,000
tra, trb
psiA
psiB
30,000
40,000
antibiotics resistance
plasmid replication
stable inheritance
SOS inhibition
conjugal transfer
transposases
others
hypothetical
replicon sequence in PlasmidFinder
ST32 C3
pO145_122715_2
48,572 bp
40,000
10,000
30,000
20,000
replication
regulator
phage morphogenesis
lysis
others
hypothetical
tRNAs

Fig S4. Plasmids found in the completely sequenced O145:H28 strains.
(A) Dot plot presentation of the sequence similarity (>99% sequence identity) of the Vir plasmids of six completely sequenced O145:H28 strains. The Vir plasmids of strains RM9872 (C2, accession no. CP028380), 122715 (C3, no. AP019709), 112648 (C5, no. AP019707), RM13514 (C6, no. CP006028), and RM13516 (C7, no. CP006263) were compared with that of strain 10942 (C1, no. AP019704) using BLASTN. The Vir plasmid of the ST6130 strain (C7) was highly divergent in sequence from the ST32 plasmids. (B) Comparison of the Vir plasmid of the O145:H28 strain 10942 (C1) belonging to ST32 with that of the O145:H28 strain RM13516 (C7) belonging to ST6130 and that of the O26:H11 strain 11368 (no. AP010954). The levels of nucleotide sequence identities between CDSs are shown by heatmap. The "plasmid-related" CDSs include those for several plasmid-related functions such as conjugation, partitioning, and SOS inhibition. The GenomeMatcher software was used for BLASTN analyses and displaying the results in panels A and B. (C) The dot plot matrixes of the concatenated sequences of the four NV plasmids each found in the four completely sequenced O145:H28 strains; strains 10942, 122715, RM13514 (no. CP006029) and RM13516 (no. CP006264) are shown. Sequences were compared using the GenomeMatcher software with 90% identity threshold. Several small segments are shared by the C1 and C6 NV plasmids in a region indicated by a magenta-colored rectangle. An enlarged view of this region is also shown. The types of plasmid replicons of each plasmid are indicated in parentheses (N.D., not detected by PlasmidFinder). (D) Circular maps of the NV plasmids found in the two strains sequenced in this study are shown; that of strain 10942 (no. AP019705) and that of strain 122715(no. AP019710).

Supplemental Table S1. *E.coli* O145:H28 strains analyzed in this study.

Isolation status							Genome sequence status				
Strain	ST[*1]/clade[*2]	*stx* subtype	Country	Host or Source	Patient symptoms (D, BD, HUS)	Year	Levels	Total length (bp)	No. of scaffolds/contigs	Accession number	Reference
CFSAN026805	32/A	*stx1a*/*stx2a*	USA	animals	NI	1999	draft	5,259,212	199	LDCU01000001-199	[8]
CFSAN026804	32/A	*stx1a*/*stx2a*	USA	human	NI	1999	draft	5,235,609	193	LGZD01000001-193	[8]
EH1904	32/A	*stx2c*	BEL	human	BD	2010	draft	5,367,273	185	BJPG01000001-185	This study
2015C-3125	32/A	*stx1a*/*stx2a*	USA	human	NI	2014	complete[*3]	5,604,464	3	CP027763-5	[9]
14040	32/A	*stx1a*/*stx2a*	JPN	human	BD	2014	draft	5,589,674	418	BJQW01000001-418	This study
14744	32/A	*stx1a*/*stx2a*	JPN	human	D	2014	draft	5,285,611	269	BJSA01000001-269	This study
PNUSAE001375	32/A	*stx1a*/*stx2a*	USA	human	NI	2015	draft	5,379,338	308	SRX1248093	from the NCBI DB
PNUSAE002278	32/A	*stx1a*/*stx2a*	USA	human	NI	2016	draft	5,397,728	356	SRX1614965	from the NCBI DB
KSU166	32/A	*stx1a*/*stx2a*	USA	human	NI	N/A	draft	5,226,701	185	SRX3841830	from the NCBI DB
FSIS1500875	32/B	*stx1a*	USA	foods	NI	2013	draft	5,337,063	224	SRX1117195	from the NCBI DB
FSIS1501198	32/B	*stx1a*	USA	animals	NI	2015	draft	5,383,203	268	SRX1715745	from the NCBI DB
FSIS1503305	32/B	*stx1a*	USA	animals	NI	2015	draft	5,286,465	238	SRX1727555	from the NCBI DB
MOD1-EC1641	32/B	*stx1a*	USA	human	NI	1988	draft	5,216,531	304	SRX2207756	from the NCBI DB
CFSAN026848	32/B	*stx1a*	USA	animals	NI	2013	draft	5,233,639	172	LHAB01000001-172	[8]
12E115	32/B	*stx1a*/*stx2a*	JPN	human	BD	2012	draft	5,502,662	220	BJPU01000001-220	This study
499	32/B	*stx1a*/*stx2a*	JPN	human	asymptomatic	2008	draft	5,322,234	223	BJQM01000001-223	This study
699	32/B	*stx1a*	JPN	human	BD	2011	draft	5,403,681	225	BJQN01000001-225	This study
10942	32/B	*stx1a*/*stx2a*	JPN	human	BD	2011	complete	5,538,172	3	AP019703-5	This study
133313	32/B	*stx1a*	JPN	human	D	2012	draft	5,569,505	404	BJQS01000001-404	This study
140385	32/B	*stx1a*/*stx2a*	JPN	human	BD	2013	draft	5,486,387	449	BJQT01000001-449	This study
142179	32/B	*stx1a*	JPN	human	BD	2014	draft	5,309,703	375	BJRE01000001-375	This study
142245	32/B	*stx1a*/*stx2a*	JPN	human	asymptomatic	2014	draft	5,380,388	513	BJRF01000001-513	This study
150811	32/B	*stx1a*	JPN	human	asymptomatic	2013	draft	5,387,706	365	BJRR01000001-365	This study
150814	32/B	*stx1a*	JPN	human	D	2014	draft	5,477,504	346	BJRS01000001-346	This study
150952	32/B	*stx1a*/*stx2a*	JPN	human	BD	2015	draft	5,549,290	336	BJRT01000001-336	This study
151349	32/B	*stx1a*/*stx2a*	JPN	human	BD	2014	draft	5,406,502	401	BJRV01000001-401	This study
151733	32/B	*stx1a*/*stx2a*	JPN	human	BD	2015	draft	5,392,950	362	BJRX01000001-362	This study
160509	32/B	*stx1a*	JPN	human	D	2015	draft	5,381,015	344	BJSJ01000001-344	This study
PNUSAE001424	32/B	*stx1a*	USA	human	NI	2015	draft	5,287,951	234	SRX1272274	from the NCBI DB
PNUSAE001425	32/B	*stx2a*	USA	human	NI	2015	draft	5,293,769	234	SRX1272275	from the NCBI DB
PNUSAE002248	32/B	*stx1a*	USA	human	NI	2012	draft	5,282,225	376	SRX1590518	from the NCBI DB
PNUSAE002551	32/B	*stx1a*	USA	human	NI	2012	draft	5,358,950	258	SRX1701493	from the NCBI DB
PNUSAE003373	32/B	*stx1a*	USA	human	NI	2013	draft	5,359,175	352	SRX1939209	from the NCBI DB
2010C-3162	32/C	*stx1a*/*stx2a*	USA	human	NI	2010	draft	5,255,693	308	SRX730371	from the NCBI DB
2014C-4056	32/C	*stx1a*/*stx2a*	USA	human	NI	2014	draft	5,334,961	227	SRX730658	from the NCBI DB
PNUSAE000230	32/C	*stx1a*/*stx2a*	USA	human	NI	2015	draft	5,370,994	234	SRX869250	from the NCBI DB
PNUSAE000273	32/C	*stx1a*/*stx2a*	USA	human	NI	2008	draft	5,437,182	220	SRX886075	from the NCBI DB
PNUSAE000277	32/C	*stx1a*/*stx2a*	USA	human	NI	2008	draft	5,295,755	255	SRX886082	from the NCBI DB
PNUSAE000265	32/C	*stx1a*	USA	human	NI	2012	draft	5,451,835	210	SRX894085	from the NCBI DB
PNUSAE000267	32/C	*stx1a*/*stx2a*	USA	human	NI	2012	draft	5,456,233	213	SRX894087	from the NCBI DB
PNUSAE000270	32/C	*stx1a*/*stx2a*	USA	human	NI	2013	draft	5,416,609	234	SRX894090	from the NCBI DB
PNUSAE000272	32/C	*stx1a*/*stx2a*	USA	human	NI	2013	draft	5,544,591	231	SRX894091	from the NCBI DB
PNUSAE001283	32/C	*stx1a*/*stx2a*	USA	human	NI	2015	draft	5,372,148	324	SRX1248156	from the NCBI DB
PNUSAE000097	32/C	*stx1a*/*stx2a*	USA	human	NI	2014	draft	5,411,334	259	SRX1295165	from the NCBI DB
132418	32/C	*stx1a*	JPN	human	BD	2013	draft	5,290,283	208	BJQL01000001-208	This study
RM9872	32/C	*stx2a*	USA	animals	NI	2009	complete	5,475,442	2	CP028379-80	[10]
2014C-3084	32/C	*stx1a*/*stx2a*	USA	human	NI	2013	draft	5,586,933	4	CP027319-22	[9]
16003	32/C	*stx1a*/*stx2a*	JPN	human	asymptomatic	2016	draft	5,384,645	262	BJSM01000001-262	This study
PNUSAE000897	32/C	*stx1a*/*stx2a*	USA	human	NI	2015	draft	5,372,340	223	SRX1272484	from the NCBI DB
PNUSAE001434	32/C	*stx1a*/*stx2a*	USA	human	NI	2015	draft	5,356,405	218	SRX1301453	from the NCBI DB
PNUSAE001746	32/C	*stx2a*	USA	human	NI	2015	draft	5,319,852	198	SRX1471477	from the NCBI DB
PNUSAE001714	32/C	*stx2a*	USA	human	NI	2015	draft	5,323,250	193	SRX1471500	from the NCBI DB
PNUSAE001900	32/C	*stx1a*/*stx2a*	USA	human	NI	N/A	draft	5,324,566	233	SRX1541848	from the NCBI DB
PNUSAE001520	32/C	*stx1a*/*stx2a*	USA	human	NI	2015	draft	5,377,136	252	SRX1541921	from the NCBI DB
2014C-3105	32/C	*stx1a*/*stx2a*	USA	human	NI	2013	draft	5,337,918	189	SRX1594668	from the NCBI DB
KSU165	32/C	*stx1a*	USA	human	NI	N/A	draft	5,322,193	252	SRX3841832	from the NCBI DB
FSIS1504619	32/D	*stx1a*	USA	foods	NI	2015	draft	5,303,863	244	SRX1386649	from the NCBI DB
MOD1-EC6137	32/D	*stx1a*	USA	animals	NI	1983	draft	5,246,835	380	SRX1991250	from the NCBI DB
PNUSAE003246	32/D	*stx1a*	USA	human	NI	2016	draft	5,356,138	218	SRX1828787	from the NCBI DB
PNUSAE003283	32/D	*stx1a*	USA	human	NI	2016	draft	5,392,570	288	SRX1874969	from the NCBI DB
PNUSAE003289	32/D	*stx1a*	USA	human	NI	2016	draft	5,346,113	327	SRX1874973	from the NCBI DB
2015-10-218 E	32/D	*stx1a*	USA	animals	NI	2015	draft	5,326,063	221	SRX3841831	from the NCBI DB
PNUSAE000466	32/E	*stx2a*	USA	human	NI	2015	draft	5,421,540	204	SRX986531	from the NCBI DB
PNUSAE000494	32/E	*stx2c*	USA	human	NI	2015	draft	5,272,079	233	SRX1037189	from the NCBI DB
FSIS1505314	32/E	*stx2c*	USA	foods	NI	2015	draft	5,339,409	251	SRX1534642	from the NCBI DB
FSIS1503307	32/E	*stx2c*	USA	human	NI	2015	draft	5,318,248	316	SRX1727571	from the NCBI DB
2012C-4474	32/E	*stx2a*	USA	human	NI	2012	draft	5,330,659	224	SRX347702	from the NCBI DB
2012C-4477	32/E	*stx2a*	USA	human	NI	2012	draft	5,308,360	215	SRX347703	from the NCBI DB
2012C-4480	32/E	*stx2a*	USA	human	NI	2012	draft	5,301,179	230	SRX347706	from the NCBI DB
5044	32/E	*stx2c*	JPN	human	asymptomatic	2001	draft	5,351,828	175	BJPC01000001-175	This study
09E126	32/E	*stx2a*	JPN	human	asymptomatic	2010	draft	5,246,165	160	BJPR01000001-160	This study
12E070	32/E	*stx2a*	JPN	human	BD	2012	draft	5,408,754	188	BJPS01000001-188	This study
12E109	32/E	*stx2a*	JPN	human	BD	2012	draft	5,448,273	211	BJPT01000001-211	This study
110509	32/E	*stx2a*	JPN	human	BD	2010	draft	5,328,432	181	BJPW01000001-181	This study
111430	32/E	*stx2a*	JPN	human	BD	2011	draft	5,302,274	185	BJPX01000001-185	This study
112312	32/E	*stx2a*	JPN	human	BD	2011	draft	5,425,291	189	BJPZ01000001-189	This study
112716	32/E	*stx2a*	JPN	human	BD	2011	draft	5,428,605	202	BJQA01000001-202	This study
120517	32/E	*stx2a*	JPN	human	BD	2011	draft	5,395,458	198	BJQE01000001-198	This study
121427	32/E	*stx1a*	JPN	human	BD	2012	draft	5,344,121	220	BJQF01000001-220	This study
122200	32/E	*stx2a*	JPN	human	BD	2012	draft	5,467,448	198	BJQG01000001-198	This study
122715	32/E	*stx2a*	JPN	human	BD/HUS	2012	complete	5,554,407	3	AP019708-10	This study
130296	32/E	*stx2a*	JPN	human	BD	2012	draft	5,579,873	194	BJQH01000001-194	This study
131713	32/E	*stx2a*	JPN	human	BD	2013	draft	5,333,843	206	BJQI01000001-206	This study
131990	32/E	*stx2a*	JPN	human	BD	2013	draft	5,319,215	189	BJQK01000001-189	This study
PV11-35	32/E	*stx2a*	JPN	human	asymptomatic	2011	draft	5,515,179	222	BJQO01000001-222	This study
PV11-59	32/E	*stx2a*	JPN	human	BD	2011	draft	5,256,396	217	BJQP01000001-217	This study
PV11-77	32/E	*stx2a*	JPN	human	D	2011	draft	5,364,930	217	BJQQ01000001-217	This study
EC3283	32/E	*stx2a*	JPN	human	BD	2011	draft	5,333,988	200	BJQR01000001-200	This study
140933	32/E	*stx2a*	JPN	human	BD	2014	draft	5,473,770	565	BJQX01000001-565	This study
141500	32/E	*stx2a*	JPN	human	BD	2014	draft	5,399,454	559	BJRB01000001-559	This study
142095	32/E	*stx2a*	JPN	human	BD	2014	draft	5,375,308	444	BJRD01000001-444	This study
142527	32/E	*stx2a*	JPN	human	BD	2014	draft	5,316,486	297	BJRI01000001-297	This study
142664	32/E	*stx2a*	JPN	human	asymptomatic	2014	draft	5,379,057	333	BJRK01000001-333	This study
150316	32/E	*stx2a*	JPN	human	D	2014	draft	5,219,026	241	BJRO01000001-241	This study
150317	32/E	*stx2a*	JPN	human	BD	2014	draft	5,186,409	366	BJRP01000001-366	This study
151109	32/E	*stx2a*	JPN	human	BD	2015	draft	5,487,311	557	BJRU01000001-557	This study
151747	32/E	*stx2a*	JPN	human	BD	2015	draft	5,381,811	354	BJRY01000001-354	This study
151836	32/E	*stx2a*	JPN	human	BD	2015	draft	5,405,340	322	BJRZ01000001-322	This study
152767	32/E	*stx2a*	JPN	human	D	2015	draft	5,428,297	272	BJSF01000001-272	This study
153152	32/E	*stx1a*	JPN	human	asymptomatic	2015	draft	5,326,786	350	BJSG01000001-350	This study
160674	32/E	*stx2a*	JPN	human	BD	2016	draft	5,270,910	270	BJSK01000001-270	This study
161161	32/E	*stx2a*	JPN	human	D	2016	draft	5,323,161	277	BJSO01000001-277	This study
161318	32/E	*stx2a*	JPN	human	BD	2016	draft	5,313,480	226	BJSP01000001-226	This study
161676	32/E	*stx2a*	JPN	human	asymptomatic	2016	draft	5,375,572	235	BJSQ01000001-235	This study
162442	32/E	*stx2a*	JPN	human	BD	2016	draft	5,364,186	294	BJST01000001-294	This study
PNUSAE001216	32/E	*stx2c*	USA	human	NI	2015	draft	5,345,406	386	SRX1142450	from the NCBI DB
2012C-4214	32/E	*stx2a*	USA	human	NI	2012	draft	5,393,583	187	SRX1587021	from the NCBI DB
2014C-3190	32/E	negative	USA	human	NI	2013	draft	5,339,133	258	SRX1637512	from the NCBI DB
2014C-3327	32/E	*stx2d*	USA	human	NI	2013	draft	5,468,887	259	SRX1659931	from the NCBI DB
PNUSAE002850	32/E	*stx2c*	USA	human	NI	2015	draft	5,511,624	286	SRX1715872	from the NCBI DB
PNUSAE002955	32/E	*stx2a*	USA	human	NI	2016	draft	5,383,536	286	SRX1741389	from the NCBI DB
PNUSAE000258	32/F	*stx2a*	USA	human	NI	2010	draft	5,447,635	226	SRX886076	from the NCBI DB
PNUSAE000263	32/F	*stx1a*	USA	human	NI	2011	draft	5,340,402	221	SRX894083	from the NCBI DB
2013C-3293	32/F	negative	USA	human	NI	2012	draft	5,295,498	202	SRX1011158	from the NCBI DB
PNUSAE00098	32/F	*stx1a*	USA	human	NI	2014	draft	5,379,671	272	SRX1301635	from the NCBI DB
FSIS1502550	32/F	*stx1a*	USA	foods	NI	2015	draft	5,362,219	220	SRX1589723	from the NCBI DB
FSIS1502976	32/F	*stx1a*	USA	human	NI	2015	draft	5,265,314	188	SRX1589725	from the NCBI DB
173758	32/F	*stx1a*	GBR	human	NI	2015	draft	5,462,433	319	SRX1797089	from the NCBI DB
03-6430	32/F	*stx1a*	CAN	human	NI	2014	draft	5,369,481	210	LEAB00000001-210	[11]
12188	32/F	*stx1a*	JPN	human	asymptomatic	2007	draft	5,388,292	185	BJOZ01000001-185	This study
1380	32/F	*stx1a*	JPN	human	NI	1998	draft	5,382,993	233	BJPA01000001-233	This study
1649	32/F	*stx1a*	JPN	human	asymptomatic	1998	draft	5,454,299	229	BJPB01000001-229	This study
8100	32/F	*stx1a*	JPN	human	asymptomatic	2003	draft	5,397,403	177	BJPD01000001-177	This study

9793	32/F	*stx1a*	JPN	human	D	2005	draft	5,414,155	218	BJPE01000001-218	This study
EH1910	32/F	*stx2a*	BEL	human	D	2010	draft	5,479,003	259	BJPH01000001-259	This study
EH1995	32/F	*stx1a*	BEL	foods	NI	2010	draft	5,294,665	194	BJPI01000001-194	This study
EH2197	32/F	*stx1a*	BEL	human	BD	2012	draft	5,328,179	223	BJPM01000001-223	This study
EH2201	32/F	*stx2a*	BEL	human	HUS	2012	draft	5,422,499	230	BJPN01000001-230	This study
EH2246	32/F	*stx2a*	BEL	human	HUS	2013	draft	5,332,058	239	BJPO01000001-239	This study
EH1992	32/F	*stx1a*	BEL	foods	NI	2010	draft	5,292,610	216	BJPP01000001-216	This study
111698	32/F	*stx1a*	JPN	human	BD	2011	draft	5,325,755	205	BJPY01000001-205	This study
112648	32/F	*stx1a/stx2a*	JPN	human	BD	2011	complete	5,579,570	2	AP019706-7	This study
112808	32/F	*stx1a/stx2d*	JPN	human	BD	2011	draft	5,391,812	244	BJQB01000001-244	This study
120412	32/F	*stx1a*	JPN	human	BD	2011	draft	5,317,204	226	BJQD01000001-226	This study
131719	32/F	*stx1a*	JPN	human	BD	2013	draft	5,337,867	184	BJQJ01000001-184	This study
95-3192	32/F	*stx2a*	USA	clinical	NI	N/A	complete	5,385,516	1	CP027362	[9]
141262	32/F	*stx1a*	JPN	human	BD	2014	draft	5,380,321	419	BJQZ01000001-419	This study
142526	32/F	*stx1a*	JPN	human	BD	2014	draft	5,429,155	552	BJRH01000001-552	This study
142612	32/F	*stx1a*	JPN	human	asymptomatic	2014	draft	5,356,137	380	BJRJ01000001-380	This study
150034	32/F	*stx1a*	JPN	human	BD	2014	draft	5,273,839	455	BJRL01000001-455	This study
150149	32/F	*stx1a/stx2a*	JPN	human	BD	2014	draft	5,354,787	339	BJRM01000001-339	This study
152282	32/F	*stx1a*	JPN	human	BD	2015	draft	5,273,151	291	BJSB01000001-291	This study
152517	32/F	*stx1a*	JPN	human	BD	2015	draft	5,342,346	346	BJSE01000001-346	This study
162405	32/F	*stx1a*	JPN	human	D	2016	draft	5,382,844	280	BJSS01000001-280	This study
162594	32/F	*stx1a*	JPN	human	asymptomatic	2016	draft	5,387,253	240	BJSU01000001-240	This study
PNUSAE002018	32/F	*stx1a*	USA	human	NI	2012	draft	5,382,310	377	SRX1561961	from the NCBI DB
PNUSAE001966	32/F	*stx2a*	USA	human	NI	2015	draft	5,325,211	336	SRX1562052	from the NCBI DB
PNUSAE001969	32/F	*stx2a*	USA	human	NI	2015	draft	5,309,532	221	SRX1562055	from the NCBI DB
PNUSAE002250	32/F	*stx2a*	USA	human	NI	2015	draft	5,397,176	344	SRX1590519	from the NCBI DB
2013C-4497	32/F	*stx1a*	USA	human	NI	2013	draft	5,402,162	263	SRX1599927	from the NCBI DB
PNUSAE002645	32/F	*stx2a*	USA	human	NI	2016	draft	5,398,467	227	SRX1733495	from the NCBI DB
PNUSAE000738	32/G	*stx2a*	USA	human	NI	2014	draft	5,398,786	223	SRX1305472	from the NCBI DB
241810	32/G	negative	GBR	human	NI	2016	draft	5,291,605	277	SRX1793482	from the NCBI DB
241761	32/G	*stx2a*	GBR	human	NI	2016	draft	5,308,521	350	SRX1793510	from the NCBI DB
132030	32/G	*stx2a*	GBR	human	NI	2015	draft	5,278,508	295	SRX1795568	from the NCBI DB
130322	32/G	*stx2a*	GBR	human	NI	2015	draft	5,278,766	317	SRX1796216	from the NCBI DB
173582	32/G	*stx2a*	GBR	human	NI	2015	draft	5,382,143	303	SRX1797196	from the NCBI DB
FHI95	32/G	*stx2a*	NOR	human	NI	2010	draft	5,175,895	124	CCRD00000001-134	[12]
FHI82	32/G	*stx2a*	NOR	human	BD	2009	draft	5,529,476	47	LM996757-LM996803	[12]
STEC1442	32/G	*stx2a*	NLD	human	BD	2014	draft	5,369,955	170	LOGK00000001-170	[13]
4865/96	32/G	*stx2a*	DEU	human	NI	1996	draft	5,220,130	345	AGTL00000001-345	from the NCBI DB
STEC731	32/G	*stx2a*	NLD	human	D	2013	draft	5,251,140	170	LOFN01000001-170	[13]
EH1846	32/G	*stx2a*	BEL	human	BD	2009	draft	5,347,600	237	BJPF01000001-237	This study
EH2011	32/G	*stx2a*	BEL	human	BD	2011	draft	5,379,469	193	BJPJ01000001-193	This study
EH2085	32/G	*stx2a*	BEL	human	BD	2011	draft	5,358,269	243	BJPK01000001-243	This study
EH2086	32/G	*stx2a*	BEL	human	HUS	2011	draft	5,364,897	225	BJPL01000001-225	This study
09E025	32/G	*stx2a*	JPN	human	BD	2009	draft	5,541,177	201	BJPQ01000001-201	This study
112991	32/G	*stx2a*	JPN	human	BD	2011	draft	5,291,602	194	BJQC01000001-194	This study
95	32/G	*stx2a*	JPN	human	asymptomatic	2014	draft	5,622,467	468	BJRC01000001-468	This study
16002	32/G	*stx2a*	JPN	human	BD	2016	draft	5,236,402	258	BJSN01000001-258	This study
PNUSAE001494	32/G	*stx2a*	USA	human	NI	2015	draft	5,427,404	243	SRX1309273	from the NCBI DB
PNUSAE002415	32/G	*stx2a*	USA	human	NI	N/A	draft	5,417,344	231	SRX1653106	from the NCBI DB
PNUSAE002493	32/G	*stx2c*	USA	human	NI	N/A	draft	5,342,920	384	SRX1709510	from the NCBI DB
PNUSAE002883	32/G	*stx2a*	USA	human	NI	2016	draft	5,300,226	182	SRX1733464	from the NCBI DB
PNUSAE003213	32/G	*stx2a*	USA	human	NI	2016	draft	5,344,723	219	SRX1837222	from the NCBI DB
2014C-3004	32/G	*stx1a*	USA	human	NI	2013	draft	5,354,443	218	SRX1926637	from the NCBI DB
VL1495n	32/G	negative	NZL	animals	NI	2016	draft	5,226,866	287	SRX3294556	from the NCBI DB
VL1287n	32/G	negative	NZL	animals	NI	2016	draft	5,330,426	358	SRX3294534	from the NCBI DB
82EZXG	32/G	*stx2a*	GBR	human	D	2015	draft	5,283,767	427	SRX3421138	from the NCBI DB
2014C-3016	32/H	*stx1a*	USA	human	NI	2014	draft	5,445,567	362	SRX730487	from the NCBI DB
PNUSAE000139	32/H	*stx2a*	USA	human	NI	2014	draft	5,658,338	442	SRX826410	from the NCBI DB
PNUSAE000274	32/H	*stx2a*	USA	human	NI	2008	draft	5,696,569	274	SRX886078	from the NCBI DB
PNUSAE000275	32/H	*stx2a*	USA	human	NI	2008	draft	5,472,081	254	SRX886080	from the NCBI DB
PNUSAE000276	32/H	*stx2a*	USA	human	NI	2008	draft	5,668,642	270	SRX886081	from the NCBI DB
PNUSAE000261	32/H	*stx2a*	USA	human	NI	2011	draft	5,445,153	268	SRX894081	from the NCBI DB
PNUSAE000262	32/H	*stx2a*	USA	human	NI	2011	draft	5,467,882	283	SRX894082	from the NCBI DB
PNUSAE000264	32/H	*stx2a*	USA	human	NI	2012	draft	5,497,781	298	SRX894084	from the NCBI DB
PNUSAE000266	32/H	*stx2a*	USA	human	NI	2012	draft	5,507,907	291	SRX894086	from the NCBI DB
PNUSAE000268	32/H	*stx2a*	USA	human	NI	2012	draft	5,466,687	252	SRX894088	from the NCBI DB
PNUSAE000269	32/H	*stx1a/stx2a*	USA	human	NI	2013	draft	5,475,754	319	SRX894089	from the NCBI DB
PNUSAE000259	32/H	*stx2a*	USA	human	NI	2010	draft	5,741,972	293	SRX913333	from the NCBI DB
FSIS1503258	32/H	*stx2a*	USA	animals	NI	2014	draft	5,468,837	291	SRX1122551	from the NCBI DB
PNUSAE001370	32/H	*stx2a*	USA	human	NI	2015	draft	5,471,571	239	SRX1248088	from the NCBI DB
PNUSAE001336	32/H	*stx2a*	USA	human	NI	2015	draft	5,462,427	257	SRX1248138	from the NCBI DB
PNUSAE001339	32/H	*stx2a*	USA	human	NI	2015	draft	5,559,972	306	SRX1248141	from the NCBI DB
PNUSAE001340	32/H	*stx2a*	USA	human	NI	2015	draft	5,640,630	362	SRX1248142	from the NCBI DB
PNUSAE001342	32/H	*stx2a*	USA	human	NI	2015	draft	5,553,186	332	SRX1248144	from the NCBI DB
PNUSAE001285	32/H	*stx2a*	USA	human	NI	2015	draft	5,466,427	285	SRX1248158	from the NCBI DB
PNUSAE000889	32/H	*stx2a*	USA	human	NI	2013	draft	5,382,632	288	SRX1272475	from the NCBI DB
FSIS1605420	32/H	*stx2a*	USA	foods	NI	2015	draft	5,623,993	358	SRX1534641	from the NCBI DB
FSIS1605419	32/H	*stx2a*	USA	foods	NI	2015	draft	5,612,498	331	SRX1534643	from the NCBI DB
PNUSAE000756	32/H	*stx2a*	USA	human	NI	2014	draft	5,430,683	307	SRX1772444	from the NCBI DB
MOD1-EC5842	32/H	negative	USA	animals	NI	1983	draft	5,336,160	286	SRX1988695	from the NCBI DB
RM13514	32/H	*stx2a*	USA	human	NI	2010	complete	5,737,294	3	CP006027-9	[14]
StOlav172	32/H	*stx2a*	NOR	human	BD	2012	draft	5,562,109	239	JZDY01000001-239	[15]
CFSAN026846	32/H	*stx2a*	USA	animals	NI	2013	draft	5,427,855	237	LGZI01000001-237	[8]
12E129	32/H	*stx2a*	JPN	human	BD	2013	draft	5,458,196	248	BJPV01000001-248	This study
IB14005	32/H	*stx2a*	JPN	human	D	2014	draft	5,496,386	525	BJQU01000001-525	This study
141115	32/H	*stx2a*	JPN	human	D	2014	draft	5,634,174	497	BJQY01000001-497	This study
140166	32/H	*stx2a*	JPN	human	BD	2014	draft	5,568,417	481	BJRA01000001-481	This study
150222	32/H	*stx1a*	JPN	human	D	2014	draft	5,517,690	630	BJRN01000001-630	This study
Ech14022	32/H	*stx2a*	JPN	human	BD	2014	draft	5,407,725	349	BJRQ01000001-349	This study
H27V05	32/H	*stx2a*	JPN	human	BD	2015	draft	5,634,447	470	BJRW01000001-470	This study
EC3734	32/H	*stx2a*	JPN	human	BD	2015	draft	5,638,456	363	BJSC01000001-363	This study
152489	32/H	*stx2a*	JPN	human	BD	2015	draft	5,436,133	313	BJSD01000001-313	This study
160018	32/H	*stx2a*	JPN	human	D	2015	draft	5,442,475	308	BJSH01000001-308	This study
KIH15-140	32/H	*stx2a*	JPN	human	D	2015	draft	5,582,205	345	BJSI01000001-345	This study
PNUSAE000631	32/H	*stx2a*	USA	human	NI	2015	draft	5,516,756	289	SRX1053602	from the NCBI DB
PNUSAE001624	32/H	*stx2a*	USA	human	NI	2015	draft	5,371,636	325	SRX1471562	from the NCBI DB
PNUSAE001606	32/H	*stx2a*	USA	human	NI	2015	draft	5,451,288	253	SRX1478490	from the NCBI DB
PNUSAE002446	32/H	*stx2a*	USA	human	NI	2016	draft	5,555,362	267	SRX1628904	from the NCBI DB
2010C-3515	32/H	*stx2a*	USA	human	NI	2010	draft	5,552,488	249	SRX1659897	from the NCBI DB
2010C-3522	32/H	*stx2a*	USA	human	NI	2010	draft	5,496,903	228	SRX1659899	from the NCBI DB
PNUSAE002575	32/H	*stx2a*	USA	human	NI	2016	draft	5,360,187	342	SRX1701487	from the NCBI DB
PNUSAE002440	32/H	*stx2a*	USA	human	NI	2015	draft	5,497,632	365	SRX1701512	from the NCBI DB
PNUSAE003020	32/H	*stx2a*	USA	human	NI	2011	draft	5,513,357	422	SRX1770373	from the NCBI DB
PNUSAE003447	32/H	*stx2a*	USA	human	NI	2016	draft	5,557,942	346	SRX1884239	from the NCBI DB
PNUSAE003371	32/H	*stx2a*	USA	human	NI	2013	draft	5,578,582	406	SRX1939207	from the NCBI DB
FSIS1700607	SLV of ST32/H	*stx2a*	USA	foods	NI	2017	draft	5,441,923	256	SRX2846814	from the NCBI DB
OLC0719	32/H	*stx1a*	CAN	NI	NI	2007	draft	5,538,385	371	SRX3266773	from the NCBI DB
PNUSAE011367	32/H	*stx2a*	USA	clinical	NI	2017	draft	5,415,460	246	SRX3478615	from the NCBI DB
PARC449	137	negative	CAN	NI	NI	2014	draft	5,428,942	181	LEAC00000001-181	[11]
AA053	137	negative	DNK	human	NI	2012	draft	5,294,101	155	ERX1089266	from the NCBI DB
MOD1-EC6028	137	*stx2a*	USA	animals	NI	1983	draft	5,461,670	396	SRX1989428	from the NCBI DB
FHI84	137	*stx1a*	NOR	human	BD	2010	draft	5,357,753	166	CCVQ01000001-176	[12]
FHI87	137	*stx1a*	NOR	human	asymptomatic	2009	draft	5,527,716	42	LM996976-79, LM996981-7018	[12]
StOlav173	137	*stx2a*	NOR	human	NI	2012	draft	5,630,790	222	JZDZ01000001-222	[15]
RM13516	6130	*stx2a*	BEL	human	NI	2007	complete	5,559,008	3	CP006262-4	[14]
RM12761	6130	*stx2a*	BEL	foods	NI	2007	complete*4	5,559,014	3	CP007133-5	[16]

*1 Sequence types (STs) were determined by using the MLST Database from Warwick University based on the gene sequences for *adk*, *fumC*, *gyrB*, *icd*, *mdh*, *purA*, and *recA*.

*2 Determined for the ST32 strains by hierBAPS.

*3 Incomplete genome sequence of the plasmid (CP027764) has been deposited.

*4 Strain RM12561 was excluded from the detailed analysis because its genome is nearly identical to that of RM13516.

SLV, Single locus variant

NI, no information

D, Diarrhea; BD, Bloody Diarrhea; HUS, Hemolytic Uremic Syndrome

Supplemental Table S3. Phage genomes used for constructing the in-house phage DB used in this study.

	accession_number	strain or host	phage name
**Prophages identified in this study**			
	AP019703	*Escherichia coli* O145:H28 str. 10942	10942_P01-10942_P17
	AP019706	*Escherichia coli* O145:H28 str. 112648	122715_P01-122715_P16
	AP019708	*Escherichia coli* O145:H28 str. 122715	122715_P01-122715_P16
	CP027763	*Escherichia coli* O145:H28 str. 2015C-3125	2015C-3125_P01-2015C-3125_P18
	CP028379	*Escherichia coli* O145:H28 str. RM9872	RM9872_P01-RM9872_P16
	CP027362	*Escherichia coli* O145:H28 str. 95-3192	95-3192_P01-95-3192_P16
	CP006027	*Escherichia coli* O145:H28 str. RM13514	RM13514_P01-RM13514_P20
	CP006262	*Escherichia coli* O145:H28 str. RM13516	RM13516_P01-RM13516_P12
**Prophages accurately annotated in the genomes we previously sequenced**			
	BA000007	*Escherichia coli* O157:H7 str. Sakai	Sp1-Sp18
	AP010953	*Escherichia coli* O26:H11 str. 11368	ECO26_P01-ECO26_P21
	AP010958	*Escherichia coli* O103:H2 str. 12009	ECO103_P01-ECO103_P15
	AP010960	*Escherichia coli* O111:H- str. 11128	ECO111_P01-ECO111_P17
	AB426048-64	*Escherichia coli* O111:H- str. B171-8	GEI0.58, 0.81, 1.03, 1.18, 1.31, 1.79, 1.94, 1.98, 2.04, 2.21, 2.43, 2.69, 4.26
	FM180568	*Escherichia coli* O127:H6 str. E2348/69	PP_1-PP_13
	AP013063	*Serratia marcescens* str. SM39	PP1-PP7
	HG326223	*Serratia marcescens* str. Db11	PP1-PP2
**Phage genomes in public DB**			
	NC_000866.4	*Escherichia coli*	Enterobacteria phage T4 (T4)
	NC_000902.1	*Escherichia coli* O157:H7	Enterobacteria phage VT2-Sakai (Bacteriophage VT2-Sa)
	NC_000924.1	*Escherichia coli* O157:H7 EDL933	Enterobacteria phage 933W
	NC_000929.1	*Escherichia coli*	Enterobacteria phage Mu
	NC_001271.1	*Yersinia enterocolitica* (type O:3)	Yersinia phage phiYeO3-12
	NC_001330.1	*Escherichia coli*	Enterobacteria phage alpha3
	NC_001332.1	*Escherichia coli*	Enterobacteria phage I2-2
	NC_001416.1	*Escherichia coli*	Enterobacteria phage lambda
	NC_001417.2	*Escherichia coli*	Enterobacteria phage MS2
	NC_001420.2	*Escherichia coli*	Enterobacteria phage G4 sensu lato
	NC_001421.2	*Escherichia coli*	Enterobacteria phage PRD1
	NC_001422.1	*Escherichia coli*	Enterobacteria phage phiX174 sensu lato
	NC_001426.1	*Escherichia coli*	Enterobacteria phage BZ13
	NC_001604.1	*Escherichia coli*	Enterobacteria phage T7 (T7)
	NC_001609.1	*Escherichia coli*	Enterobacteria phage P4
	NC_001890.1	*Escherichia coli*	Enterobacteria phage Qbeta
	NC_001895.1	*Escherichia coli*	Enterobacteria phage P2
	NC_001901.1	*Escherichia coli*	Enterobacteria phage N15
	NC_001954.1	*Escherichia coli*	Enterobacteria phage If1
	NC_002014.1	*Escherichia coli*	Enterobacteria phage Ike
	NC_002166.1	*Escherichia coli*	Enterobacteria phage HK022
	NC_002167.1	*Escherichia coli*	Enterobacteria phage HK97
	NC_002371.2	*Salmonella enterica* serovar typhimurium	Enterobacteria phage P22
	NC_002730.1	*Escherichia coli*	Salmonella phage HK620
	NC_003287.2	*Escherichia coli*	Enterobacteria phage M13
	NC_003298.1	*Escherichia coli*	Enterobacteria phage T3
	NC_003356.1	*Escherichia coli*	Enterobacteria phage phiP27
	NC_003444.1	*Shigella flexneri*	Enterobacteria phage SfV
	NC_003525.1	*Escherichia coli* O157:H7	Stx2 converting phage I (Stx2 converting bacteriophage I)
	NC_004301.1	*Escherichia coli*	Enterobacteria phage FI sensu lato
	NC_004313.1	*Salmonella* Typhimurium	Salmonella phage ST64B
	NC_004348.1	*Salmonella* Typhimurium	Salmonella phage ST64T
	NC_004745.1	*Yersinia pestis*	Yersinia phage L-413C
	NC_004775.1	*Salmonella enterica*	Salmonella phage epsilon15
	NC_004777.1	*Yersinia pestis*	Yersinia phage phiA1122
	NC_004813.1	*Escherichia coli*	Enterobacteria phage BP-4795
	NC_004831.2	*Salmonella* Typhimurium	Enterobacteria phage SP6
	NC_004913.2	*Escherichia coli* O157:H7	Escherichia Stx1 converting bacteriophage (Stx1 converting
	NC_004914.2	*Escherichia coli* O157:H7	Stx2 converting phage II (Stx2 converting bacteriophage II)
	NC_004928.1	*Escherichia coli*	Enterobacteria phage RB69 (RB69)
	NC_005066.1	*Escherichia coli*	Enterobacteria phage RB49 (RB49)
	NC_005069.1	*Yersinia enterocolitica*	Yersinia phage PY54
	NC_005282.1	*Salmonella*	Salmonella phage Felix01
	NC_005340.1	*Salmonella*	Enterobacteria phage PsP3
	NC_005344.1	*Shigella flexneri*	Shigella phage Sf6
	NC_005833.1	*Escherichia coli*	Enterobacteria phage T1
	NC_005841.1	*Salmonella* typhimurium DT104	Enterobacteria phage ST104
	NC_005856.1	*Escherichia coli*	Enterobacteria phage P1
	NC_005857.1	*Klebsiella oxytoca*	Klebsiella phage phiKO2
	NC_005859.1	*Escherichia coli*	Enterobacteria phage T5
	NC_006940.2	unknown	Salmonella phage SS3e
	NC_006949.1	*Salmonella* typhimurium	Enterobacteria phage ES18
	NC_007023.1	Enterobacteriaceae	Enterobacteria phage RB43 (RB43)
	NC_007291.1	*Escherichia coli* O157:H7	Enterobacteria phage JK06
	NC_007456.1	*Escherichia coli*	Enterobacteria phage K1F
	NC_007603.1	*Escherichia coli*	Enterobacteria phage RTP
	NC_007637.1	*Escherichia coli*	Enterobacteria phage K1E
	NC_007804.2	*Escherichia coli* O157:H7	Escherichia phage phiV10
	NC_007817.1	*Escherichia coli*	Enterobacteria phage ID2 Moscow/ID/2001
	NC_007821.1	*Escherichia coli*	Enterobacteria phage WA13 sensu lato
	NC_007856.1	*Escherichia coli*	Enterobacteria phage ID18 sensu lato
	NC_008152.1	*Escherichia coli*	Enterobacteria phage K1-5
	NC_008464.1	*Escherichia coli*	Stx2-converting phage 86 (Stx2-converting bacteriophage 8)
	NC_008515.1	Enterobacteriaceae	Enterobacteria phage RB32
	NC_008694.1	*Yersinia pestis*	Yersinia phage Berlin
	NC_008720.1	*Escherichia coli*	Escherichia phage N4
	NC_009014.1	*Erwinia amylovora* NCPPB 595	Enterobacteria phage Era103
	NC_009232.1	*Salmonella* enteritidis	Salmonella phage SETP3
	NC_009514.1	*Escherichia coli*	Enterobacteria phage cdtI (Bacteriophage cdtI)
	NC_009540.1	*Escherichia coli* B	Enterobacteria phage TLS
	NC_009821.1	*Escherichia coli* K-12 (F+)	Enterobacteria phage Phi1
	NC_010105.1	*Escherichia coli*	Enterobacteria phage JS98
	NC_010106.1	*Escherichia coli*	Enterobacteria phage phiEcoM-GJ1
	NC_010237.1	*Escherichia coli* O157:H7 str. Min27	Enterobacteria phage Min27
	NC_010324.1	*Escherichia coli*	Enterobacteria phage Phieco32
	NC_010391.1	*Salmonella* typhimurium LT2	Salmonella phage Fels-1
	NC_010392.1	*Salmonella* typhimurium LT2	Phage Gifsy-1
	NC_010393.1	*Salmonella* typhimurium LT2	Phage Gifsy-2
	NC_010463.1	*Salmonella* typhimurium LT2	Salmonella phage Fels-2
	NC_010495.1	*Salmonella* enterica	Salmonella phage Vi II-E1
	NC_010583.1	*Escherichia coli*	Enterobacteria phage EPS7
	NC_010807.1	*Salmonella* spp.	Salmonella phage phiSG-JL2

NC_011038.1	*Yersinia pestis*	Yersinia phage Yepe2
NC_011040.1	*Escherichia coli*	Enterobacteria phage BA14
NC_011041.1	*Escherichia coli*	Escherichia phage rv5
NC_011042.1	*Escherichia coli*	Enterobacteria phage EcoDS1
NC_011043.1	*Klebsiella* sp. 390	Klebsiella phage K11
NC_011045.1	*Escherichia coli*	Enterobacteria phage 13a
NC_011085.2	*Morganella morganii*	Morganella phage MmP1
NC_011356.1	*Escherichia coli* O157:H7	Enterobacteria phage YYZ-2008
NC_011357.1	*Escherichia coli* O157:H7	Stx2-converting phage 1717
NC_011534.1	*Kluyvera cryocrescens*	Kluyvera phage Kvp1
NC_011802.1	*Salmonella enterica*	Salmonella phage SE1
NC_011811.1	*Erwinia amylovora*	Erwinia phage phiEa21-4
NC_011976.1	*Salmonella enterica* subsp. *enterica* serovar Anatum	Salmonella phage epsilon34
NC_012223.1	*Escherichia coli*	Enterobacteria phage SSL-2009a
NC_012635.1	unknown	Enterobacteria phage RB51
NC_012638.1	unknown	Enterobacteria phage RB14
NC_012740.1	*Escherichia coli* K12	Enterobacteria phage JSE
NC_012741.1	*Escherichia coli* K12	Enterobacteria phage JS10
NC_012749.1	*Escherichia coli* O157:H7	Escherichia phage wV8
NC_012868.1	*Escherichia coli* strain K12	Enterobacteria phage St-1
NC_013059.1	*Salmonella enterica* subsp. *enterica* serovar Anatum	Salmonella phage g341c
NC_013594.1	*Escherichia coli*	Escherichia phage D108
NC_013647.1	*Klebsiella pneumoniae*	Klebsiella phage KP32
NC_013649.2	*Klebsiella pneumoniae*	Klebsiella phage KP34
NC_013693.1	*Shigella boydii*	Shigella phage phiSboM-AG3
NC_014036.1	*Klebsiella pneumoniae*	Klebsiella phage KP15
NC_014260.1	*Escherichia coli* strain 8099	Enterobacteria phage IME08
NC_014467.1	*Escherichia coli*	Enterobacteria phage RB16
NC_014595.1	*Shigella sonnei*	Shigella phage SP18
NC_014662.1	*Escherichia coli*	Enterobacteria phage CC31
NC_014792.1	*Escherichia coli*	Enterobacteria phage vB_EcoM-VR7
NC_014900.1	*Salmonella enterica* serovar *Typhimurium*	Salmonella phage ST160
NC_015249.1	*Escherichia coli*	Enterobacteria phage 285P
NC_015269.1	*Salmonella Typhimurium* ; *Escherichia coli*	Enterobacteria phage SPC35
NC_015271.1	*Salmonella enterica* subsp. *enterica* serovar Typhi	Salmonella phage Vi06
NC_015292.1	unknown	Erwinia phage phiEa104
NC_015295.1	unknown	Erwinia phage phiEt88
NC_015296.1	*Salmonella enterica* subsp. *enterica* serovar Typhi	Salmonella phage Vi01
NC_015456.1	*Shigella flexneri*	Shigella phage Shfl1
NC_015457.1	*Shigella flexneri*	Shigella phage Shfl2
NC_015585.1	*Pantoea agglomerans*	Pantoea phage LIMEzero
NC_015719.1	unknown	Enterobacteria phage K30
NC_015938.1	unknown	Salmonella phage 7-11
NC_016071.1	*Salmonella enteritidis*	Salmonella phage PVP-SE1
NC_016073.1	*Salmonella* sp. *and Escherichia coli* O157	Salmonella phage SFP10
NC_016158.1	*Escherichia coli*	Escherichia phage HK639
NC_016160.1	*Escherichia coli K-12*	Escherichia phage HK75
NC_016163.1	*Yersinia enterocolitica*	Yersinia phage phiR1-37
NC_016566.1	*Shigella sonnei*	Shigella phage EP23
NC_016570.1	*Escherichia coli* ; O157:H7; NCTC 12900	Escherichia phage vB_EcoM_CBA120
NC_016761.1	pathenogenic *Salmonella* bacterium	Salmonella phage SPN1S
NC_016763.1	*Salmonella enterica* serovar Enteritidis	Salmonella phage SE2
NC_016767.1	*Erwinia pyrifoliae*	Erwinia phage PEp14
NC_017969.1	*Escherichia coli*	Escherichia phage bV_EcoS_AKFV33
NC_017974.1	*Cronobacter sakazakii*	Cronobacter phage CR3
NC_017985.1	*Salmonella* sp.	Salmonella phage SPN9CC
NC_018275.1	*Salmonella* serovar Haardt	Salmonella phage vB_SemP_Emek
NC_018279.1	*Salmonella* serovar Winston	Salmonella phage vB_SosS_Oslo
NC_018454.1	UV-induced *Cronobacter sakazakii* ES15	Cronobacter phage phiES15
NC_018832.1	*Providencia stuartii* isolate MRSN 2154	Providencia phage Redjac
NC_018835.1	*Escherichia coli* DE172	Enterobacteria phage NJ01
NC_018837.1	unknown	Pectobacterium phage My1
NC_018843.1	unknown	Salmonella phage SSU5
NC_018846.1	*Escherichia coli* CB13374, serotype O104:H4	Escherichia phage P13374
NC_018854.1	*Escherichia coli* KBP21	Escherichia phage KBNP21
NC_018855.1	*Escherichia coli* DM01	Enterobacteria phage HX01
NC_018859.1	*Escherichia coli* KBP135	Escherichia phage KBNP135
NC_019398.1	*Cronobacter sakazakii*	Cronobacter phage vB_CsaM_GAP161
NC_019399.1	*Escherichia coli*	Enterobacteria phage vB_EcoM_ACG-C40
NC_019400.1	*Cronobacter sakazakii*	Cronobacter phage vB_CsaM_GAP31
NC_019401.1	*Cronobacter sakazakii*	Cronobacter phage vB_CsaM_GAP32
NC_019402.1	*Cronobacter sakazakii*	Cronobacter phage vB_CsaP_GAP52
NC_019403.1	*Escherichia coli*	Enterobacteria phage vB_EcoP_ACG-C91
NC_019404.1	*Escherichia coli*	Enterobacteria phage vB_EcoS_ACG-M12
NC_019417.1	*Salmonella* sp.	Salmonella phage SPN19
NC_019419.1	*Escherichia coli* O157:H7	Enterobacteria phage JL1
NC_019420.1	unknown	Edwardsiella phage KF-1
NC_019423.1	*Escherichia coli*	Enterobacter phage IME11
NC_019442.1	unknown	Escherichia phage TL-2011c
NC_019445.1	unknown	Escherichia phage TL-2011b
NC_019452.1	*Escherichia coli* O157:H7	Escherichia phage PhaxI
NC_019454.1	*Pantoea agglomerans*	Pantoea phage LIMElight
NC_019485.1	*Enterobacter cloacae*	Enterobacter phage EcP1
NC_019488.1	unknown	Salmonella phage RE-2010
NC_019500.1	*Escherichia coli* O78.A	Enterobacteria phage Bp7
NC_019501.1	*Escherichia coli*	Enterobacteria phage IME10
NC_019503.1	*Escherichia coli*	Enterobacteria phage ime09
NC_019504.1	*Erwinia amylovora*	Erwinia phage vB_EamM-Y2
NC_019505.1	*Escherichia coli*	Escherichia phage wV7
NC_019509.1	*Cronobacter sakazakii* ATCC29544	Cronobacter phage ESP2949-1
NC_019510.1	*Erwinia amylovora*	Erwinia phage vB_EamP-L1
NC_019514.1	*Erwinia amylovora*	Erwinia phage vB_EamP-S6
NC_019517.1	*Escherichia coli*	Enterobacteria phage vB_EcoM-FV3
NC_019520.1	*Escherichia* sp.	Escherichia phage phiKT
NC_019522.1	*Pectobacterium carotovorum*	Pectobacterium phage ZF40 (vB_PcaM-ZF40)
NC_019524.1	*Enterobacter cancerogenus*	Enterobacter phage Enc34
NC_019526.1	*Klebsiella sp.*	Enterobacteria phage vB_KleM-RaK2
NC_019530.1	*Salmonella typhimurium*	Salmonella phage PhiSH19
NC_019539.1	unknown	Salmonella phage vB_SenS-Ent1
NC_019542.1	unknown	Pectobacterium phage PP1
NC_019545.1	*Salmonella typhimurium*	Salmonella phage SPN3UB
NC_019704.1	*Escherichia coli*	Enterobacteria phage mEp237
NC_019705.1	*Escherichia coli*	Enterobacteria phage mEpX2
NC_019706.1	*Escherichia coli*	Enterobacteria phage mEp043 c-1

NC_019707.1	*Escherichia coli* J53 (plasmid RIP69)	Enterobacteria phage M
NC_019708.1	*Escherichia coli*	Enterobacteria phage mEp235
NC_019709.1	*Escherichia coli*	Enterobacteria phage mEpX1
NC_019710.1	*Escherichia coli*	Enterobacteria phage HK140
NC_019711.1	*Escherichia coli*	Enterobacteria phage HK629
NC_019714.1	*Escherichia coli*	Enterobacteria phage HK446
NC_019715.1	*Escherichia coli*	Enterobacterial phage mEp234
NC_019716.1	*Escherichia coli*	Enterobacteria phage mEp460
NC_019717.1	*Escherichia coli*	Enterobacteria phage HK225
NC_019718.1	*Escherichia coli*	Enterobacteria phage vB_EcoS_Rogue1
NC_019719.1	*Escherichia coli*	Enterobacteria phage HK633
NC_019720.1	*Escherichia coli*	Enterobacterial phage mEp213
NC_019721.1	*Escherichia coli*	Enterobacterial phage mEp390
NC_019723.1	*Escherichia coli*	Enterobacteria phage HK630
NC_019724.1	*Escherichia coli*	Enterobacteria phage HK578
NC_019725.1	unknown	Escherichia phage ADB-2
NC_019767.1	*Escherichia coli*	Enterobacteria phage HK544
NC_019768.1	*Escherichia coli*	Enterobacteria phage HK106
NC_019769.1	*Escherichia coli*	Enterobacteria phage HK542
NC_019781.1	*Klebsiella pneumoniae*	Klebsiella phage KP36
NC_019909.1	*Yersinia enterocolitica*	Yersinia phage phiR1-RT
NC_019910.1	unknown	Salmonella phage SKML-39
NC_019911.1	*Yersinia enterocolitica*	Yersinia phage phi80-18
NC_019919.1	*Yersinia enterocolitica*	Yersinia phage phiR201
NC_019920.1	*Enterobacteriaceae*	Enterobacteria phage C-1 INW-2012
NC_019922.1	*Enterobacteriaceae*	Enterobacteria phage Hgal1
NC_019926.1	unknown	Erwinia phage phiEa100
NC_019927.1	*Cronobacter sakazakii*	Cronobacter phage ENT47670
NC_019929.1	*Erwinia amylovora*	Erwinia phage phiEaH2
NC_019932.1	*Erwinia amylovora*	Erwinia phage ENT90
NC_019934.1	*Cronobacter sakazakii*	Cronobacter phage ENT39118
NC_020083.1	*Serratia* sp.	Serratia phage phiMAM1
KC333879.1	*Escherichia coli*	Enterobacteria phage phiJLA23
KC295538.1	*Escherichia coli* O157	Escherichia phage PBECO 4
KC237308.1	*Escherichia coli* C	Enterobacteria phage ID204 Moscow/ID
KC206276.1	*Escherichia coli* O78:K80	Escherichia phage EC1
JQ015307.1	*Pectobacterium atrosepticum* SCRI1043	Pectobacterium phage phiTE
JF461087.1	*Salmonella enterica*	Salmonella phage FO1a
JX000007.1	*Yersinia pestis*	Yersinia phage R
JX866719.1	*Klebsiella pneumoniae* JDM777	Klebsiella phage JD001
KC107834.1	*Cronobacter sakazakii* strain 51329	Cronobacter phage vB_CskP_GAP227
AB767244.1	unknown	Edwardsiella phage MSW-3
JX871397.1	*Escherichia coli*	Enterobacteria phage phi80 (Lula)
AB757801.1	unknown	Edwardsiella phage IW-1
HQ918180.1	*Klebsiella pneumoniae*	Klebsiella phage KP27
HE858210.2	unknown	Enterobacteria phage RB43 (RB43)
HE981739.1	unknown	Enterobacteria phage RB43 (RB43)
JX913857.1	*Escherichia coli* C	synthetic Enterobacteria phage phiX174.1f
JX561091.1	*Escherichia coli*	Escherichia phage phAPEC8
JX181825.1	unknown	Salmonella phage STML-198
JX560968.1	*Escherichia coli* O157:H7	Escherichia phage EC6
JX181824.1	unknown	Salmonella phage SSE-121
JQ966307.1	*Escherichia coli*	Enterobacterio phage MS2
JX412914.1	*Escherichia coli*	Enterobacteria phage M13
AP011113.1	*Escherichia coli* O157:H7	Enterobacteria phage AR1
HM208303.1	*Escherichia coli* O157:H7	Stx2 converting phage vB_EcoP_24B
HE956711.1	*Yersinia pestis*	Yersinia phage phiD1
HE956707.1	*Yersinia enterocolitica*	Yersinia phage phiR8-01
JX128258.1	*Escherichia coli* O157:H7	Escherichia phage ECML-117
JX202565.1	unknown	Salmonella phage wksl3
JX128259.1	*Escherichia coli* O157:H7	Escherichia phage ECML-134
JX128257.1	*Escherichia coli* O157:H7	Escherichia phage ECML-4
GU903191.1	*Escherichia coli*	Escherichia phage vB_EcoM_ECO1230-10
FR775895.2	*Escherichia coli* K92	Enterobacteria phage phi92
HQ665011.1	*Escherichia coli*	Escherichia phage bV_EcoS_AKFV33
HE600015.1	*Dickeya solani*	Dickeya phage vB_DsoM_LIMEstone1
JN641803.1	unknown	Salmonella phage SPN3US
JF314845.1	*Cronobacter sakazakii*	Cronobacter phage ES2
JN600960.1	*Escherichia coli*	Enterobacteria phage IME10
JF770475.1	*Escherichia coli* CFT073	Escherichia phage phiEB49
HQ728263.1	*Erwinia amylovora*	Erwinia phage vB_EamM-M7
HQ110083.1	*Cronobacter sakazakii*	Cronobacter phage ESSI-2
HQ406778.1	*Salmonella Typhimurium* ; *Escherichia coli*	Enterobacteria phage SPC35
FJ000341.1	*Salmonella enterica* subsp. *enterica* serovar Anatum	Salmonella phage g341c
AM084414.1	unknown	Enterobacteria phage K1F
FQ482084.1	unknown	Erwinia phage phiEa1H
FM180578.1	*E. coli O157:H7* strain CB2851	Enterobacteria phage 2851
AM084415.1	unknown	Enterobacteria phage K1E
AP005154.1	*Escherichia coli* O157:H7 Morioka V526	Stx2 converting phage II
HQ333270.1	*Yersinia pestis*	Yersinia phage Yep-phi
GU196280.1	*Escherichia coli* O18:K1:H7	Escherichia phage K1-ind(2)
GU196281.1	*Escherichia coli* O18:K1:H7	Escherichia phage K1-ind(3)
GU196277.1	*Escherichia coli* O18:K1:H7	Escherichia phage K1-dep(4)
GU196279.1	*Escherichia coli* O18:K1:H7	Escherichia phage K1-ind(1)
GU196278.1	*Escherichia coli* O18:K1:H7	Escherichia phage K1-dep(1)
AF503408.1	unknown	Enterobacteria phage P7
HM137666.1	*Escherichia coli*	Enterobacteria phage T4T
AY598820.1	unknown	VCSM13 interference-resistant helper phage
CP000711.1	*Escherichia coli* RS218	Enterobacteria phage CUS-3
EU078592.1	*Escherichia coli* BL21(DE3)	Enterobacteria phage DE3
AY848688.1	unknown	Enterobacteria phage PR772
AY848686.1	unknown	Enterobacteria phage PR4
AY848684.1	unknown	Enterobacteria phage L17
AY848687.1	unknown	Enterobacteria phage PR5
AY848685.1	unknown	Enterobacteria phage PR3
J02451.1	unknown	Enterobacteria phage fd
AP005153.1	*Escherichia coli* O157:H7 Morioka V526	Escherichia Stx1 converting bacteriophage
AB255436.1	*Escherichia coli* O86:H-, strain DIJ1	Stx2-converting phage 86
AP004402.1	*Escherichia coli* O157:H7 Okayama O-27	Stx2 converting phage I
AB102868.1	*Salmonella typhimurium* DT104	Enterobacteria phage ST104
DQ085810.2	unknown	Enterobacteria phage alpha3
AY135486.1	unknown	Enterobacteria phage PsP3
NC_000867.1	*Pseudoalteromonas*	Pseudoalteromonas phage PM2
NC_001396.1	*Xanthomonas campestris*	Xanthomonas phage Cf1c

NC_001697.1	*Haemophilus influenzae*	Haemophilus phage HP1
NC_001956.1	*Vibrio cholerae* O139	Vibrio phage fs2 (Vibrio cholerae filamentous bacteriophage fs-2)
NC_002362.1	*Vibrio parahaemolyticus* O3:K6	Vibrio phage VfO3K6 (Bacteriophage VfO3K6)
NC_002363.1	*Vibrio parahaemolyticus*	Vibrio phage VfO4K68 (Bacteriophage VfO4K68)
NC_002700.2	*Acinetobacter* genomosp. 16	Acinetobacter phage AP205
NC_003313.1	*Vibrio cholerae* O139	Vibrio phage K139
NC_003315.1	*Haemophilus influenzae*	Haemophilus phage HP2
NC_003327.2	*Vibrio cholerae* O139	Vibrio phage VSK
NC_003907.2	*Vibrio parahaemolyticus*	Vibrio phage VpV262
NC_004306.1	*Vibrio cholerae* O139	Vibrio phage fs1 (Vibrio cholerae O139 fs1 phage)
NC_004456.1	*Vibrio harveyi*	Vibrio phage VHML
NC_004736.1	*Vibrio cholerae*	Vibrio phage VGJphi
NC_004902.1	*Xanthomonas oryzae*	Xanthomonas phage Xp10
NC_005083.2	*Vibrio parahaemolyticus*	Vibrio phage KVP40
NC_005135.1	*Aeromonas salmonicida*	Aeromonas phage 44RR2.8t
NC_005260.1	*Aeromonas hydrophila*	Aeromonas phage Aeh1 (Aeh1)
NC_005879.1	*Vibrio cholerae*	Vibrio phage VP2
NC_005891.1	*Vibrio cholerae*	Vibrio phage VP5
NC_005948.1	*Vibrio parahaemolyticus*	Vibrio phage Vf33 (Bacteriophage Vf33)
NC_005949.1	*Vibrio parahaemolyticus*	Vibrio phage Vf12 (Bacteriophage Vf12)
NC_006294.1	*Vibrio cholerae*	Vibrio phage KSF-1phi
NC_006953.1	*Listonella pelagia*	Listonella phage phiHSIC
NC_007022.1	*Aeromonas salmonicida*	Aeromonas phage 31
NC_007024.1	*Xanthomonas campestris* pv. *pelargonii*	Xanthomonas phage Xp15
NC_007149.1	*Vibrio*	Vibriophage VP4
NC_007189.1	*Stenotrophomonas maltophilia*	Stenotrophomonas phage phiSMA9
NC_007709.1	*Xanthomonas oryzae*	Xanthomonas phage OP1
NC_007710.1	*Xanthomonas oryzae*	Xanthomonas phage OP2
NC_007902.1	*Sodalis glossinidius* str. morsitans	Sodalis phage phiSG1
NC_008193.1	*Pasteurella multocida*	Pasteurella phage F108
NC_008201.1	*Mannheimia haemolytica*	Mannheimia phage phiMHaA1
NC_008208.1	*Aeromonas salmonicida*	Aeromonas phage 25
NC_009016.1	*Vibrio parahaemolyticus*	Vibrio phage VP882
NC_009542.2	*Aeromonas media*	Aeromonas phage phiO18P
NC_009543.1	*Xanthomonas oryzae*	Xanthomonas phage Xop411
NC_009990.1	*Thalassomonas loyana* LMG 22536	Thalassomonas phage BA3
NC_010275.1	*Vibrio cholerae*	Vibrio phage kappa
NC_010342.1	*Halomonas aquamarina*	Halomonas phage phiHAP-1
NC_011589.1	*Stenotrophomonas maltophilia*	Stenotrophomonas phage S1
NC_012662.1	*Vibrio parahaemolyticus* PMC57.5	Vibrio phage VP93
NC_012742.1	*Xanthomonas campestris* pv. campestris	Xanthomonas phage phiL7
NC_012757.1	*Vibrio cholerae*	Vibrio phage VEJphi
NC_013597.1	*Aggregatibacter actinomycetemcomitans* D11S-1	Aggregatibacter phage S1249
NC_013599.1	*Xylella fastidiosa*	Xylella phage Xfas53
NC_013600.1	*Sodalis glossinidius*	Sodalis phage SO-1
NC_013651.1	unknown	Vibrio phage N4
NC_014635.1	*Aeromonas salmonicida*	Aeromonas phage phiAS4
NC_014636.1	*Aeromonas salmonicida*	Aeromonas phage phiAS5
NC_014660.1	unknown	Acinetobacter phage Ac42
NC_014661.1	*Acinetobacter johnsonii*	Acinetobacter phage Acj61
NC_014663.1	*Acinetobacter johnsonii*	Acinetobacter phage Acj9
NC_015157.1	*Vibrio cholerae*	Vibrio phage ICP1
NC_015158.1	*Vibrio cholerae*	Vibrio phage ICP2
NC_015159.1	*Vibrio cholerae*	Vibrio phage ICP3
NC_015208.1	unknown	Pseudomonas phage phi15
NC_015209.1	*Vibrio cholerae* KMN002	Vibrio phage CTX
NC_015250.1	*Acinetobacter johnsonii*	Acinetobacter phage 133
NC_015251.1	*Aeromonas salmonicida*	Aeromonas phage 65
NC_015272.1	unknown	Pseudomonas phage KPP10
NC_015293.1	*Pseudoalteromonas* sp. H105	Pseudoalteromonas phage H105/1
NC_015586.1	*Stenotrophomonas maltophilia*	Stenotrophomonas phage phiSHP2
NC_015933.1	unknown	Escherichia phage vB_EcoP_G7C
NC_016162.1	*Vibrio cholerae* str. 10E09PW02	Vibrio phage VCY-phi
NC_016567.1	*Vibrio* sp. SWAT-3	Vibrio phage SIO-2
NC_017981.1	*Xanthomonas vesicatoria*	Xanthomonas phage vB_XveM_DIBBI
NC_017984.1	*Acinetobacter baumannii*	Acinetobacter bacteriophage AP22
NC_018087.1	unknown	Acinetobacter phage ZZ1
NC_018088.1	*Colwellia psychrerythaea* strain 34H	Colwellia phage 9A
NC_018269.1	*Marinomonas* sp. IMCC12026	Marinomonas phage P12026
NC_019416.1	*Stenotrophomonas maltophilia*	Stenotrophomonas phage IME15
NC_019455.1	*Haemophilus parasuis* 34086b	Haemophilus phage SuMu
NC_019457.1	*Vibrio cholerae* ElTor	Vibrio phage CP-T1 (vB_VchM-CP-T1)
NC_019518.1	*Vibrio cholerae*	Vibrio phage vB_VchM-138
NC_019527.1	*Aeromonas salmonicida*	Aeromonas phage vB_AsaM-56 (phage 56-Popoff)
NC_019528.1	*Aeromonas salmonicida* subsp. *salmonicida*	Aeromonas phage phiAS7
NC_019529.1	*Vibrio parahaemolyticus* ATCC 33844	Vibrio phage pVp-1
NC_019538.1	*Aeromonas hydrophila*	Aeromonas phage CC2
NC_019540.1	*Salinivibrio costicola*	Salinivibrio phage CW02
NC_019541.1	*Acinetobacter baumannii* YMC/09/02/B1251	Acinetobacter phage Bphi-B1251
NC_019543.1	*Aeromonas salmonicida*	Aeromonas phage Aes508
NC_019713.1	*Vibrio parahaemolyticus*	Vibrio phage vB_VpaS_MAR10
NC_019722.1	*Vibrio parahaemolyticus*	Vibrio phage vB_VpaM_MAR
NC_019923.1	unknown	Pseudomonas phage AF
NC_019933.1	*Xanthomonas citri*	Xanthomonas phage CP1
KC311669.1	carbapenem-resistant *Acinetobacter baumannii*	Acinetobacter phage AB3
AF399011.1	unknown	Pseudomonas phage phiKZ
JX434033.1	unknown	Pseudomonas phage JBD88a
JX434031.1	unknown	Pseudomonas phage JBD24
JX434032.1	unknown	Pseudomonas phage JBD30
JX434030.1	unknown	Pseudomonas phage JBD5
JN811560.1	unknown	Pseudomonas phage JBD26
AB720064.1	*Xanthomonas citri*	Xanthomonas citri phage CP2
JX560521.1	*Acinetobacter soli* KZ-1	Acinetobacter phage phiAC-1
JX976549.1	*Acinetobacter baumannii*	Acinetobacter phage IME-AB2
JX495041.1	unknown	Pseudomonas phage JBD18
JX495042.1	unknown	Pseudomonas phage JBD25
JF270478.1	*Psychrobacter* sp. MV2	Psychrobacter phage Psymv2
JX306041.1	*Stenotrophomonas maltophilia*	Stenotrophomonas phage IME13
HM368260.1	*Acinetobacter baumannii*	Acinetobacter phage AB1
JQ801337.1	*Vibrio vulnificus*	Vibrio phage VvAW1
JN377895.1	*Aeromonas* sp.	Aeromonas phage Aes012
JQ692107.1	*Vibrio vulnificus*	Vibrio phage SSP002
JQ780163.1	*Vibrio cholerae*	Vibrio phage VP3
JF713456.1	*Vibrio harveyi* 1114GL	Vibrio phage 1

AB572858.1	*Vibrio cholerae* O139 strain ND1	Vibrio phage ND1-fs1
JN808773.1	unknown	Pseudomonas phage F_HA0480sp/Pa1651
JF712866.1	*Vibrio cholerae* O1 Classic E8021	Vibrio phage phiVC8
HQ186308.1	*Acinetobacter baumannii* M68316	Acinetobacter phage phiAB1
GU396103.1	*Aeromonas salmonicida*	Aeromonas phage PX29
DQ785801.1	*Sodalis glossinidius*	Sodalis phage phiSG1 (Sodalis plasmid pSOG3)
NC_001331.1	*Pseudomonas aeruginosa*	Pseudomonas phage Pf1
NC_001418.1	*Pseudomonas aeruginosa*	Pseudomonas phage Pf3
NC_001628.1	*Pseudomonas aeruginosa*	Pseudomonas phage PP7
NC_002484.1	*Pseudomonas aeruginosa*	Pseudomonas phage D3
NC_003278.1	*Pseudomonas aeruginosa*	Pseudomonas phage phiCTX (Pseudomonas aeruginosa phage phi CTX)
NC_004466.2	*Pseudomonas aeruginosa*	Pseudomonas phage PaP3
NC_004629.1	*Pseudomonas aeruginosa*	Pseudomonas phage phiKZ
NC_004665.1	*Pseudomonas putida*	Pseudomonas phage gh-1 (gh-1)
NC_005045.1	*Pseudomonas aeruginosa*	Enterobacteria phage phiKMV
NC_005178.1	*Pseudomonas aeruginosa*	Pseudomonas phage D3112
NC_005884.1	*Pseudomonas aeruginosa*	Pseudomonas phage PaP2
NC_006548.1	*Pseudomonas aeruginosa* PAO1 (ATCC 15692)	Pseudomonas phage B3
NC_006552.1	*Pseudomonas aeruginosa*	Pseudomonas phage F116
NC_007623.1	*Pseudomonas aeruginosa*	Pseudomonas phage EL
NC_007805.1	*Pseudomonas aeruginosa*	Pseudomonas phage F10
NC_007806.1	*Pseudomonas aeruginosa*	Pseudomonas phage 73
NC_007807.1	*Pseudomonas aeruginosa*	Pseudomonas phage 119X
NC_007808.1	*Pseudomonas aeruginosa*	Pseudomonas phage PA11
NC_007809.1	*Pseudomonas aeruginosa*	Pseudomonas phage M6
NC_007810.1	*Pseudomonas aeruginosa*	Pseudomonas phage F8
NC_008294.1	*Pseudomonas*	Pseudomonas phage PRR1 (PRR1)
NC_008717.1	*Pseudomonas aeruginosa*	Pseudomonas phage DMS3
NC_009818.1	*Pseudomonas aeruginosa* UCBPP-PA14	Pseudomonas phage MP22
NC_009935.1	*Pseudomonas aeruginosa*	Pseudomonas phage LKD16
NC_009936.1	*Pseudomonas aeruginosa*	Pseudomonas phage LKA1
NC_010116.1	*Pseudomonas aeruginosa*	Pseudomonas phage YuA
NC_010325.1	*Pseudomonas aeruginosa*	Pseudomonas phage LUZ24
NC_010326.1	*Pseudomonas aeruginosa* PAO1	Pseudomonas phage LUZ19
NC_010821.1	*Pseudomonas chlororaphis*	Pseudomonas phage 201phi2-1
NC_011105.1	*Pseudomonas aeruginosa*	Pseudomonas phage PT5
NC_011107.1	*Pseudomonas aeruginosa*	Pseudomonas phage PT2
NC_011165.1	*Pseudomonas aeruginosa*	Pseudomonas phage LBL3
NC_011166.1	*Pseudomonas aeruginosa*	Pseudomonas phage LMA2
NC_011373.1	*Pseudomonas aeruginosa*	Pseudomonas phage PAJU2
NC_011611.1	*Pseudomonas aeruginosa* PA14	Pseudomonas phage MP38
NC_011613.1	*Pseudomonas aeruginosa* PA14	Pseudomonas phage MP29
NC_011703.1	*Pseudomonas* spp.	Pseudomonas phage 14-1
NC_011756.1	*Pseudomonas aeruginosa*	Pseudomonas phage SN
NC_011810.1	*Pseudomonas aeruginosa*	Pseudomonas phage PB1
NC_012418.1	*Pseudomonas aeruginosa*	Pseudomonas phage phikF77
NC_013638.1	*Pseudomonas fluorescens* SBW25	Pseudomonas phage phi-2
NC_013691.1	*Pseudomonas aeruginosa*	Pseudomonas phage LUZ7
NC_013692.1	*Pseudomonas aeruginosa*	Pseudomonas phage LIT1
NC_015264.1	*Pseudomonas fluorescens*	Pseudomonas phage phiIBB-PF7A
NC_015294.1	*Pseudomonas aeruginosa* PAK	Pseudomonas phage PAK_P1
NC_016571.1	*Pseudomonas fluorescens* Pf1.1	Pseudomonas phage OBP
NC_016762.1	*Pseudomonas aeruginosa*	Pseudomonas phage phi297
NC_016764.1	*Pseudomonas tolaasii* LMG 2342	Pseudomonas phage Bf7
NC_016765.1	*Pseudomonas aeruginosa*	Pseudomonas phage vB_PaeS_PMG1
NC_017674.1	*Pseudomonas aeruginosa*	Pseudomonas phage JG024
NC_017864.1	*Pseudomonas aeruginosa* PA25	Pseudomonas phage vB_Pae-Kakheti25
NC_017865.1	*Pseudomonas aeruginosa* PA32	Pseudomonas phage vB_Pae-TbilisiM32
NC_017971.2	*Pseudomonas putida* PpG1	Pseudomonas phage tf
NC_017972.1	*Pseudomonas putida* var. Manila	Pseudomonas phage Lu11
NC_018274.1	*Pseudomonas aeruginosa* PA14	Pseudomonas phage MP42
NC_018282.1	*Pseudomonas aeruginosa* PAO1	Pseudomonas phage MP1412
NC_018850.1	*Pseudomonas fluorescens*	Pseudomonas phage UFV-P2
NC_019450.1	*Pseudomonas aeruginosa*	Pseudomonas phage JG004
NC_019451.1	*Pseudomonas aeruginosa*	Pseudomonas phage NH-4
NC_019813.1	*Pseudomonas aeruginosa*	Pseudomonas phage vB_PaeP_p2-10_Or1
NC_019913.1	*Pseudomonas aeruginosa* PA1	Pseudomonas phage PaP1
NC_019918.1	*Pseudomonas aeruginosa* C2-10	Pseudomonas phage vB_PaeM_C2-10_Ab1
NC_019935.1	*Pseudomonas aeruginosa*	Pseudomonas phage KPP12
KC262634.1	*Pseudomonas aeruginosa* HIM-6	Pseudomonas phage H66
JX403939.1	*Pseudomonas aeruginosa* YMC/01/01/P52	Pseudomonas phage YMC/01/01/P52_PAE_BP
HE983844.1	*Pseudomonas aeruginosa*	Pseudomonas phage vB_PaeP_C1-14_Or
JX194238.1	*Pseudomonas aeruginosa*	Pseudomonas phage PA26
JQ067084.1	*Pseudomonas aeruginosa*	Pseudomonas phage PaMx25
JQ067085.1	*Pseudomonas aeruginosa*	Pseudomonas phage PaMx73
JQ067083.1	*Pseudomonas aeruginosa*	Pseudomonas phage PaMx13
JQ307387.1	*Pseudomonas aeruginosa* PA25	Pseudomonas phage vB_Pae-Kakheti25
JN254801.1	*Pseudomonas aeruginosa*	Pseudomonas phage MR299-2
HM624080.1	*Pseudomonas aeruginosa*	Pseudomonas phage PA1/KOR/2010
HQ711985.1	*Pseudomonas aeruginosa*	Pseudomonas phage vB_PaeS_PMG1
JN175269.1	*Pseudomonas tolaasii*	Pseudomonas phage phi_Pto-bp6g
HQ630627.1	*Pseudomonas aeruginosa* strain PAO1	Pseudomonas phage PhiPA3
HM173081.1	*Pseudomonas aeruginosa*	Pseudomonas phage P3_CHA
HM173082.1	*Pseudomonas aeruginosa*	Pseudomonas phage PAK_P3
AB008550.1	*Pseudomonas aeruginosa* (strain PA158)	Pseudomonas phage phiCTX
NC_003714.1	*Pseudomonas syringae* pv. *phaseolicola*	Pseudomonas phage phi6
NC_004173.1	*Pseudomonas syringae*	Pseudomonas phage phi12
NC_004170.1	*Pseudomonas syringae*	Pseudomonas phage phi13
NC_012091.2	*Pseudomonas syringae*	Pseudomonas phage phi2954
NC_003299.1	*Pseudomonas syringae*	Pseudomonas phage phi8

Supplemental Table S4. The IS elements identified in the seven completely sequenced O145:H28 genomes.

		Copy numbers on chromosome/plasmid in						
Family	ST, Strain ID<br>Strain name	ST32, C1<br>10942	ST32, C2<br>RM9872	ST32, C3<br>122715	ST32, C4<br>95-3192[*1]	ST32, C5<br>112648	ST32, C6<br>RM13514	ST6130, C7<br>RM13516
IS*1*	IS*1F*	2/0	2/0	2/0	0	2/0	2/0	2/0
IS*1*	IS*1R*	0/0	0/0	0/0	0	0/0	0/0	1/0
IS*3*	IS*2*	1/0	1/0	1/0	2	1/0	2/0	1/0
IS*3*	IS*600*	1/4	1/4	1/4	1	1/4	9/4	2/0
IS*3*	IS*629*	37/5	39/2	32/3	34	29/2	27/5	19/3
IS*3*	IS*1203* /<br>IS*1203E*	3/1	5/2	8/1	2	6/3	3/1	0/0
IS*3*	IS*911*	1/1	1/1	1/1	1	1/1	1/1	1/0
IS*3*	IS*Cfr6*	1/0	2/0	1/0	1	1/0	1/0	0/2
IS*3*	IS*Ec16*	0/0	0/0	0/0	0	0/0	0/0	2/0
IS*3*	IS*Ec48*	0/0	0/0	0/0	0	0/0	0/0	0/1
IS*3*	IS*Kpn37*	0/0	0/0	0/0	0	0/0	0/0	0/1
IS*4*	IS*421*	0/1	0/1	0/1	0	0/1	0/1	0/0
IS*4*	IS*Ec13*	1/0	1/0	1/0	1	1/0	1/0	1/0
IS*4*	IS*Ec60*	0/0	0/0	0/0	0	0/0	0/0	1/0
IS*6*	IS*15*	0/0	0/0	0/0	0	0/0	0/1	0/0
IS*6*	IS*26*	0/1	0/0	0/0	0	0/0	0/2	0/0
IS*21*	IS*Sso4*	0/0	1/0	1/0	1	1/0	2/0	0/0
IS*30*	IS*30*	0/0	0/0	0/0	0	0/0	0/0	2/0
IS*66*	IS*682*	1/0	1/0	1/0	1	1/0	1/0	1/0
IS*66*	IS*Cro1*	1/0	1/0	1/0	1	1/0	2/0	2/0
IS*66*	IS*Ec8*	1/4	1/4	1/4	1	1/4	2/3	6/2
IS*66*	IS*Ec23*	1/0	1/0	1/0	1	1/0	1/0	7/1
IS*66*	IS*Ec47*	1/1	1/1	1/1	1	1/1	2/1	1/0
IS*66*	IS*Ec49*	0/0	1/0	1/0	1	1/0	1/0	0/0
IS*66*	IS*Ec78*	1/0	1/0	1/0	1	1/0	1/0	2/0
IS*66*	IS*Ec83*	0/4	1/4	1/4	1	1/4	1/4	2/0
IS*91*	IS*Ec84*	0/2	0/2	0/2	0	0/2	0/2	0/4
IS*91*	IS*Vsa3*	0/0	0/0	0/0	0	0/0	0/1	0/0
IS*110*	IS*Ec20*	4/0	4/0	5/0	5	4/0	8/0	6/0
IS*200* /IS*605*	IS*200C*	0/1	0/1	0/1	0	0/1	0/1	0/1
IS*200* /IS*605*	IS*609*	2/0	2/0	2/0	2	2/0	2/0	2/1
IS*256*	IS*Eic2*	0/0	0/0	0/0	0	0/0	0/0	1/0
IS*As1*	IS*Ec1*	1/0	1/0	1/0	1	1/0	1/0	1/0
IS*As1*	IS*Ec5*	1/0	1/0	1/0	1	1/0	1/0	1/0
IS*L3*	IS*Ec38*	1/1	1/1	1/1	1	1/1	1/1	1/0
	Total	62/26	70/23	66/23	61	60/24	72/28	66/16

[*1] No plasmid sequences have been deposited.

The copy numbers in C6 or C7 notably different from those of other strains are indicated by red.

Unique IS elements in C7 are indicated by cyan.

Supplemental Table S5. The PPs and IEs of seven completely sequenced O145:H28 strains.

	Integration site	ST32/B, C1 (strain 10942)				ST32/C, C2 (strain RM9872)				ST32/E, C3 (strain 122715)					
		ID	Features	Length (bp)	Virulence-related genes	ID	Features	Length (bp)	Virulence-related genes	ID	Features	Length (bp)	Virulence-related genes	ID	Features
**PPs**															
	tRNA (*argU*)	P01	lambda-like	31,700	*bor*, *lom*	P01	lambda-like	31,700	*bor*, *lom*	P01	lambda-like	31,494	*bor*, *lom*	P01	
	ybhC-*ybhB*	P02	lambda-like	34,512	*espJ*, *nleH*, *cif*, *sfpA*, *lom*, *nleG*[*1]	P02	lambda-like	34,512	*espJ*, *nleH*, *cif*, *sfpA*, *lom*, *nleG*[*1]	P02	lambda-like	35,825	*espJ*, *nleH*, *cif*, *sfpA*, *lom*, *nleG*[*1]	P02	
	cspD-*clpS*														
	tRNA (*serT*)	P03	lambda-like	28,471	*tccP*, *lom*, *espV*[*1]	P03	lambda-like	48,649	*pchA*, *tccP*, *lom*, *espV*[*1]	P03	lambda-like	48,117	*pchA*, *tccP*, *lom*, *espV*[*1]	P03	
	wrbA													P09	
	potB	P04	lambda-like	50,598	*espK*, *espN*, *espO*, *espX*, *lom*, *nleB*[*1]	P04	lambda-like	50,597	*espK*, *espN*, *espO*, *espX*, *lom*, *nleB*[*1]	P04	lambda-like	50,756	*espK*, *espN*, *espO*, *espX*, *lom*, *nleB*[*1]	P08	
	roxA-*phoQ*	P05	untypable	11,222	*pchE*	P05	untypable	11,222	*pchE*	P05	untypable	11,222	*pchE*	P07	
	ompW	P06	lambda-like	43,627	*espM*, *espO*, *nleG* x 2	P06	lambda-like	44,075	*espM*, *espO*, *nleG* x 2	P06	lambda-like	43,991	*espM*, *espO*, *nleG* x 2	P06	
	ompW	P07	lambda-like	45,405	*stx2*, *nleC*, *lom*, serine protease		lambda-like								
	ttcA	P08	lambda-like	47,683	*pchB*, *lom*, *nleC*[*1]	P07	lambda-like	55,838	*pchB*, *lom*, *nleC*[*1]	P07	lambda-like	51,230	*pchB*, *lom*, *nleC*[*1]	P05	
	ydfJ	P09	lambda-like	49,474	*nleC*, *lom*	P08	lambda-like	50,533	*lom*, *nleC*[*1]	P08	lambda-like	47,354	*pchB*, *lom*, *nleC*[*1]	P04	
	btuC-*infA*	P10	P2-like	36,529		P09	P2-like	38,003							
	yecE	P11	lambda-like	48,649	*stx1*, *nleC*, *lom*	P10	lambda-like	45,511	*stx2*, *nleC*, *lom*, serine protease						
	tRNA (*leuZ*)									P09	P2-like	36,917			
	tRNA (*serU*)	P12	lambda-like	46,169	*pchA*, *nleB*, *nleH*, *nleF*, *lom*	P11	lambda-like	46,168	*pchA*, *nleB*, *nleH*, *nleF*, *lom*	P10	lambda-like	44,680	*nleC*, *lom*	P10	lambda-like
	sbcB														
	yegQ-*yegR*														
	mlrA	P13	lambda-like	35,687	*nleA*, *lom*, *espV*[*1], *nleH*[*1]	P12	lambda-like	35,849	*nleA*, *lom*, *espV*[*1], *nleH*[*1]	P11	lambda-like	35,846	*nleA*, *lom*, *espV*[*1], *nleH*[*1]	P11	lambda-like
	tRNA (*argW*)									P12	lambda-like	62,636	*stx2*, *lom*		
	tmRNA (*ssrA*)	P14	lambda-like	10,696	*nleG*, *ospB*, *lom*	P13	lambda-like	12,006	*nleG*, *ospB*, *lom*	P13	lambda-like	29,786	*nleG*, *ospB*, *lom*	P12	lambda-like
	mscS	P15	P4-like	13,465		P14	P4-like	14,681		P14	P4-like	13,377		P13	P4-like
	yicC													P14	P4-like
	cpxP-*fleF*	P16	P2-like	33,682		P15	P2-like	33,465		P15	P2-like	33,729		P15	P2-like
	dusA														
	prfC	P17	lambda-like	29,908	*lom*	P16	lambda-like	29,908	*lom*	P16	lambda-like	29,907	*lom*	P16	
**IEs**															
	tRNA (*serX*)	IE01	SpLE1-like	82,020	*pchD, aidA-I*, *iha*, *ureA*-*G*, *terA*-*F*,*W*, *Z*	IE01	SpLE1-like	82,017	*pchD, aidA-I*, *iha*, *ureA*-*G*, *terA*-*F*,*W*, *Z*	IE01	SpLE1-like	80,129	*pchD, aidA-I*, *iha*, *ureA*-*G*, *terA*-*F*,*W*, *Z*	IE02	SpLE1-like
	tRNA (*leuZ*)	IE02		20,386		IE02		21,706		IE02		20,312		IE01	
	tRNA (*asnT*)	IE03		13,717		IE03		13,717		IE03		13,717		IE03	
	tRNA (*pheV*)	IE04		21,908		IE04		22,143		IE04		22,912		IE04	
	tRNA (*ileX*)														
	tRNA (*selC*)	IE05	LEE	46,833	T3SS machinery, *espB*, *espF*, *espG*, *espH*, *espZ*, *map*, *tir*	IE05	LEE	47,117	T3SS machinery, *espB*, *espF*, *espG*, *espH*, *espZ*, *map*, *tir*	IE05	LEE	46,977	T3SS machinery, *espB*, *espF*, *espG*, *espH*, *espZ*, *map*, *tir*	IE05	LEE
	tRNA (*pheU*)	IE06	SpLE3-like	12,900	*espL*, *nleB*, *nleE*	IE06	SpLE3-like	42,067	*espL*, *nleB*, *nleE*, *aidA*-*I*	IE06	SpLE3-like	47,716	*espL*, *nleB*, *nleE*, *aidA*-*I*	IE06	SpLE3-like
	tRNA (*leuX*)														

[*1] Pseudogene

[*2] Re-analyzed in this study

ST32/F, C4 (strain 95-3192)		ST32/F, C5 (strain 112648)				ST32/H, C6 (strain RM13514)[*2]				ST6130, C7 (strain RM13516)[*2]			
Length (bp)	Virulence-related genes	ID	Features	Length (bp)	Virulence-related genes	ID	Features	Length (bp)	Virulence-related genes	ID	Features	Length (bp)	Virulence-related genes
31,698	bor, lom	P01	lambda-like	31,700	bor, lom	P01	lambda-like	29,802	bor, lom				
34,509	espJ, nleH, sfpA, lom, cif[*1], nleG[*1]	P02	lambda-like	34,511	espJ, nleH, cif, sfpA, lom, nleG[*1]	P02	lambda-like	35,778	espJ, nleH, cif, sfpA, lom, nleG[*1]	P01	lambda-like	43,182	pchA, espJ, nleH, cif, sfpA, lom, nleG[*1]
		P03	P4-like	9,547		P03	P4-like	11,142					
49,139	pchA, nleB, nleH, nleF, espV[*1]	P04	lambda-like	47,357	pchA, tccP, lom, espV[*1]	P04	lambda-like	51,815	pchA, tccP, lom, espV[*1]	P02	lambda-like	48,928	pchA, tccP, lom, espV[*1]
64,449	stx2, bor, lom	P05	lambda-like	49,855	stx1, nleC, lom								
50,589	espK, espN, espO, espX, lom, nleB[*1]	P06	lambda-like	50,760	espK, espN, espO, espX, lom, nleB[*1]	P05	lambda-like	49,788	espK, espN, espO, espX, lom, nleB[*1]	P03	lambda-like	44,962	espK, espN, espO, espX, lom, nleB[*1]
11,222	pchE	P07	untypable	11,222	pchE	P06	untypable	11,222	pchE	P04	untypable	11,221	pchE
44,081	espM, espO, nleG x 2	P08	lambda-like	44,064	espM, espO, nleG x 2	P07	lambda-like	42,634	espM, espO, nleG x 2	P05	lambda-like	45,709	espM, espO, nleG x 2
		P09	lambda-like	44,286	stx2, nleC, lom, serine protease								
53,229	pchB, lom, nleC[*1]	P10	lambda-like	50,603	nleC, lom	P08	lambda-like	48,923	pchB, lom				
49,832	nleC, lom	P11	lambda-like	48,336	pchB, lom, nleC[*1]	P09	lambda-like	46,660	lom, nleC[*1]	P06	lambda-like	47,714	pchB, nleC, lom
		P12	lambda-like	44,286	stx2, nleC, lom, serine protease					P07	lambda-like	47,309	stx2, nleC, lom, serine protease
		P13	P2-like	37,323		P10	P2-like	25,115					
51,865	pchA, tccP, lom	P14	lambda-like	52,467	pchA, nleB, nleH, nleF, lom	P11	lambda-like	50,390	pchA, nleB, nleH, nleF, lom	P08	lambda-like	48,964	pchA, nleB, nleH, nleF, lom
						P12	lambda-like	45,388					
										P09	P2-like	35,789	
35,846	nleA, lom, espV[*1], nleH[*1]	P15	lambda-like	34,532	nleA, lom, espV[*1], nleH[*1]	P13	lambda-like	35,805	espV, nleA, lom, nleH[*1]	P10	lambda-like	57,297	espV, nleA, nleG x 2, lom, ospB[*1]
						P14	lambda-like	62,692	stx2, lom				
10,696	nleG, ospB, lom	P16	lambda-like	40,894	nleG, ospB, lom	P15	lambda-like	36,519	nleG, ospB, lom	P11	lambda-like	42,129	nleG, ospB, lom
13,367		P17	P4-like	13,368		P16	P4-like	13,368					
15,955						P17	P4-like	11,945					
33,729		P18	P2-like	33,729		P18	P2-like	33,729					
						P19	lambda-like	46,659	pchA, nleG, lom, (ospB)				
29,907	lom					P20	lambda-like	29,907	lom	P12	lambda-like	50,395	lom
81,851	pchD, aidA-I, iha, ureA-F, terA-F,W, Z	IE01	SpLE1-like	82,018	pchD, aidA-I, iha, ureA-G, terA-F,W, Z	IE01	SpLE1-like	82,235	iha, ureA-G, terA-F,W, Z	IE01	SpLE1-like	96,165	iha, ureA-G, terA-F,W, Z
20,856		IE02		19,073		IE02		13,341		IE02		14,663	
13,716		IE03		13,717		IE03		27,920		IE03		13,717	
21,951		IE04		23,452		IE04		12,470		IE04		32,050	
						IE05	SpLE1-like	65,392	espP, ureA-G, terA-F,W, Z				
46,973	T3SS machinery, espB, espF, espG, espH, espZ, map, tir	IE05	LEE	46,977	T3SS machinery, espB, espF, espG, espH, espZ, map, tir	IE06	LEE	46,695	T3SS machinery, espB, espF, espG, espH, espZ, map, tir	IE05	LEE	47,827	T3SS machinery, espB, espF, espG, espH, espZ, map, tir
44,925	espL, nleB, nleE, aidA-I	IE06	SpLE3-like	46,414	espL, nleB, nleE, aidA-I	IE07	SpLE3-like	47,676	espL, nleB, nleE, aidA-I	IE06		66,609	aidA-I
										IE07	SpLE3-like	52,073	espL, nleB, nleE, aidA-I

Supplemental Table S6. T3SS effectors and their copy numbers of the seven completely sequenced O145:H28 strains

T3SS effectors	Strains (ST, clade)						
	10942 (ST32, C1)	RM9872 (ST32, C2)	122715 (ST32, C3)	95-3192 (ST32, C4)	112648 (ST32, C5)	RM13514 (ST32, C6)*1	RM13516 (ST6130, C7)*1
EspB*2	1	1	1	1	1	1	1
EspF*2	1	1	1	1	1	1	1
EspG*2	1	1	1	1	1	1	1
EspH*2	1	1	1	1	1	1	1
EspZ*2	1	1	1	1	1	1	1
Map*2	1	1	1	1	1	1	1
Tir*2	1	1	1	1	1	1	1
EspJ	1	1	1	1	1	1	1
EspK	1	1	1	1	1	1	1
EspL	1	1	1	1	1	1	1
EspM	1	1	1	1	1	1	1
EspN	1	1	1	1	1	1	1
EspO	2	2	2	2	2	2	2
EspV	2 (2)	2 (2)	2 (2)	2 (2)	2 (2)	2 (1)	2 (1)
EspW	0	0	0	0	0	0	0
EspX	1	1	1	1	1	1	1
NleA/EspI	1	1	1	1	1	1	1
NleB	3 (1)	3 (1)	2 (1)	3 (1)	3 (1)	3 (1)	3 (1)
NleC	4 (1)	3 (1)	3 (2)	2 (1)	5 (1)	1 (1)	2
NleD	0	0	0	0	0	0	0
NleE	1	1	1	1	1	1	1
NleF	1	1	0	1	1	1	1
NleG	4 (1)	4 (1)	4 (1)	4 (1)	4 (1)	6 (1)	6 (1)
NleH	3 (1)	3 (1)	2 (1)	3 (1)	3 (1)	3 (1)	2
TccP/EspFu	1	1	1	1	1	1	1
Cif	1	1	1	1(1)	1	1	1
OspB	1	1	1	1	1	2 (1)	2 (1)
OspG	0	0	0	0	0	0	0
Total	38 (6)	36 (6)	34 (7)	35 (7)	39 (6)	38 (6)	38 (4)

Copy numbers including pseudogenes are shown. The numbers of pseudogenes are indicated in parentheses.

*1 Re-analyzed in this study.

*2 Effectors encoded in the LEE core region.

References

1. **Ogura Y, Ooka T, Iguchi A, Toh H, Asadulghani M, *et al.*** Comparative genomics reveal the mechanism of the parallel evolution of O157 and non-O157 enterohemorrhagic *Escherichia coli*. *Proc Natl Acad Sci USA* 2009;106:17939–17944. doi: 10.1073/pnas.0903585106
2. **Hayashi T, Makino K, Ohnishi M, Kurokawa K, Ishii K, *et al.*** Complete genome sequence of enterohemorrhagic *Eschelichia coli* O157:H7 and genomic comparison with a laboratory strain K-12. *DNA Res* 2001;8:11–22. doi: org/10.1093/dnares/8.1.11
3. **Ogura Y, Abe H, Katsura K, Kurokawa K, Asadulghani M, *et al.*** Systematic identification and sequence analysis of the genomic islands of the enteropathogenic *Escherichia coli* strain B171-8 by the combined use of whole-genome PCR scanning and fosmid mapping. *J Bacteriol* 2008;190:6948–6960. doi: 10.1128/JB.00625-08
4. **Iguchi A, Thomson NR, Ogura Y, Saunders D, Ooka T, *et al.*** Complete genome sequence and comparative genome analysis of enteropathogenic *Escherichia coli* O127:H6 strain E2348/69. *J Bacteriol* 2009;191:347–354, doi: 10.1128/JB.01238-08
5. **Iguchi A, Nagaya Y, Pradel E, Ooka T, Ogura Y, *et al.*** Genome evolution and plasticity of *Serratia marcescens*, an important multidrug-resistant nosocomial pathogen. *Genome Biol Evol* 2014;6:2096–2110, doi: 10.1093/gbe/evu160
6. **Kleinheinz KA, Joensen KG, Larsen MV**. Applying the ResFinder and VirulenceFinder web-services for easy identification of acquired antibiotic resistance and *E. coli* virulence genes in bacteriophage and prophage nucleotide sequences. *Bacteriophage* 2014;4:e27943, doi: 10.4161/bact.27943
7. **Ohtsubo Y, Ikeda-Ohtsubo W, Nagata Y, Tsuda M.** GenomeMatcher: A graphical user interface for DNA sequence comparison. *BMC Bioinformatics* 2008;9: 376, doi: 10.1186/1471-2105-9-376
8. **Toro M, Cao G, Rump L, Nagaraja, TG, Meng J, et al.** Genome sequences of 64 non-O157:H7 Shiga toxin-producing *Escherichia coli* strains. *Genome Announc* 2015; 3: e01067-15, doi: 10.1128/genomeA.01067-15

9. **Patel PN, Lindsey RL, Garcia-Toledo L, Rowe LA, Batra D, *et al.*** High-quality whole-genome sequences for 77 Shiga toxin-producing *Escherichia coli* strains generated with PacBio sequencing. *Genome Announc* 2018;6: e00391-18, doi: 10.1128/genomeA.00391-18

10. **Parker CT, Cooper KK, Huynh S, Smith TP, Bono JL, *et al.*** Genome sequences of eight Shiga toxin-producing *Escherichia coli* strains isolated from a produce-growing region in California. *Microbiol Resour Announc* 2018;7:e00807-18, doi: 10.1128/MRA.00807-18

11. **Mercer RG, Zheng J, Garcia-Hernandez R, Ruan L, Gänzle MG, *et al.*** Genetic determinants of heat resistance in *Escherichia coli*. *Front Microbiol* 2015;6:932, doi: 10.3389/fmicb.2015.00932

12. **Haugum K, Johansen J, Gabrielsen C, Brandal LT, Bergh K, *et al.*** Comparative genomics to delineate pathogenic potential in non-O157 Shiga toxin-producing *Escherichia coli* (STEC) from patients with and without haemolytic uremic syndrome (HUS) in Norway. *PLoS One* 2014;9: e111788, doi: 10.1371/journal.pone.0111788

13. **Ferdous M, Friedrich AW, Grundmann H, de Boer RF, Croughs PD, *et al.*** Molecular characterization and phylogeny of Shiga toxin-producing *Escherichia coli* isolates obtained from two Dutch regions using whole genome sequencing. *Clin Microbiol Infect* 2016;22: 642.e1-9, doi: 10.1016/j.cmi.2016.03.028

14. **Cooper KK, Mandrell RE, Louie JW, Korlach J, Clark TA, *et al.*** Comparative genomics of enterohemorrhagic *Escherichia coli* O145:H28 demonstrates a common evolutionary lineage with *Escherichia coli* O157:H7. *BMC Genomics* 2014;15:17, doi: 10.1186/1471-2164-15-17

15. **Gabrielsen C, Drabløs F, Afset JE.** Genome sequences of 11 Shiga toxin-producing *Escherichia coli* strains. *Genome Announc* 2015;3:e00418-15, doi: 10.1128/genomeA.00418-15

16. **Cooper KK, Mandrell RE, Louie JW, Korlach J, Clark TA, *et al.*** Complete genome sequences of two *Escherichia coli* O145:H28 outbreak strains of food origin. *Genome Announc* 2014;2: e00482-14, doi: 10.1128/genomeA.00482-14
